# Fuel types misrepresent forest structure and composition in interior British Columbia: a way forward

**DOI:** 10.1186/s42408-024-00249-z

**Published:** 2024-02-07

**Authors:** Jennifer N. Baron, Paul F. Hessburg, Marc-André Parisien, Gregory A. Greene, Sarah. E. Gergel, Lori D. Daniels

**Affiliations:** 1 Department of Forest and Conservation Sciences, Faculty of Forestry, University of British Columbia, Vancouver, BC V6T 1Z4 Canada; 2 USDA-FS, Pacific Northwest Research Station, Wenatchee, WA 98801 USA; 3 University of Washington, School of Forest and Environmental Sciences, Box 352100, Seattle, WA 98195-2100 USA; 4 Canadian Forest Service, Northern Forestry Center, Edmonton, AB T6H 3S5 Canada

**Keywords:** Wildland fire, Fuel classification, Forest inventory, Fuel mapping, Fire management, Remote sensing, Fire behavior, Canadian Fire Behavior Prediction System

## Abstract

**Background:**

A clear understanding of the connectivity, structure, and composition of wildland fuels is essential for effective wildfire management. However, fuel typing and mapping are challenging owing to a broad diversity of fuel conditions and their spatial and temporal heterogeneity. In Canada, fuel types and potential fire behavior are characterized using the Fire Behavior Prediction (FBP) System, which uses an association approach to categorize vegetation into 16 fuel types based on stand structure and composition. In British Columbia (BC), provincial and national FBP System fuel type maps are derived from remotely sensed forest inventory data and are widely used for wildfire operations, fuel management, and scientific research. Despite their widespread usage, the accuracy and applicability of these fuel type maps have not been formally assessed. To address this knowledge gap, we quantified the agreement between on-site assessments and provincial and national fuel type maps in interior BC.

**Results:**

We consistently found poor correspondence between field assessment data and both provincial and national fuel types. Mismatches were particularly frequent for (i) dry interior ecosystems, (ii) mixedwood and deciduous fuel types, and (iii) post-harvesting conditions. For 58% of field plots, there was no suitable match to the extant fuel structure and composition. Mismatches were driven by the accuracy and availability of forest inventory data and low applicability of the Canadian FBP System to interior BC fuels.

**Conclusions:**

The fuel typing mismatches we identified can limit scientific research, but also challenge wildfire operations and fuel management decisions. Improving fuel typing accuracy will require a significant effort in fuel inventory data and system upgrades to adequately represent the diversity of extant fuels. To more effectively link conditions to expected fire behavior outcomes, we recommend a fuel classification approach and emphasis on observed fuels and measured fire behavior data for the systems we seek to represent.

**Supplementary Information:**

The online version contains supplementary material available at 10.1186/s42408-024-00249-z.

## Resumen

**Antecedentes:**

Un entendimiento claro sobre la conectividad, estructura, y composición de los combustibles vegetales es esencial para un manejo efectivo de los incendios de vegetación. Sin embargo, la tipificación y mapeo de los combustibles son aspectos desafiantes debido a la amplia diversidad de las condiciones de los combustibles y su variabilidad espacial y temporal. En Canadá, los tipos de combustibles y el comportamiento potencial del fuego están caracterizados por el uso del Sistema de Predicción del Comportamiento del Fuego (Fire Behavior Prediction System, FBP), el cual usa una “aproximación asociada” para categorizar la vegetación en 16 tipos de combustibles basados en la estructura y composición de los rodales. En la Columbia Británica (BC) los mapas del sistema provincial y nacional de FBP son derivados de datos de inventarios tomados mediante sensores remotos, que son ampliamente usados para operaciones de incendios de vegetación, manejo de combustibles, e investigación científica. A pesar de su amplio uso, la exactitud y aplicabilidad de esos mapas de tipos de combustibles no han sido adecuadamente comprobadas. Para determinar este vacío en el conocimiento, cuantificamos la concordancia entre las determinaciones in situ y los mapas de combustibles provinciales y nacionales en el interior de BC.

**Resultados:**

Encontramos consistentemente una pobre correspondencia entre las determinaciones de los datos de campo y los tipos de combustibles provinciales y nacionales. Los desfasajes fueron particularmente frecuentes para: i) los ecosistemas secos del interior, ii) bosques mixtos y tipos de combustibles en bosques deciduos, y iii) condiciones de postcosecha. Para el 58% de las parcelas a campo, no hubo una concordancia adecuada entre la estructura y composición existentes. Estos desajustes fueron derivados de la exactitud y disponibilidad de datos del inventario forestal, y la baja aplicabilidad del Sistema FBP a los combustibles del interior de la Columba Británica.

**Conclusiones:**

Los desajustes en la determinación de los tipos de combustibles que nosotros identificamos pueden limitar la investigación científica, pero también es un desafío para las decisiones en las operaciones de incendios y en el manejo de los combustibles. El mejoramiento de la exactitud en la determinación de tipos de combustibles requerirá de un esfuerzo significativo en el inventario de datos y sistemas mejorados para representar adecuadamente la diversidad de los combustibles existentes. Para ligar más efectivamente las condiciones a los resultados del comportamiento, recomendamos una aproximación a la clasificación de los combustibles y énfasis en datos de los combustibles observados y del comportamiento medido para los sistemas que pretendemos representar.

## Background

Wildland surface and canopy fuels, and their contagion or connectivity, are the only elements of the fire environment that can be altered in the short term to influence future fire behavior and effects (Fernandes 2009; Thompson et al. 2013). Knowledge of the spatial pattern, structure, and composition of wildland fuels is crucial to fire management, informing fire suppression operations, wildfire risk assessments, and fuel reduction treatments (Keane et al. 2001; Keane 2015). Despite their importance, measuring and mapping wildland fuels remain notoriously challenging owing to the complexity and diversity of individual fuel components and their spatial and temporal heterogeneity (Keane et al. 2001; Arroyo et al. 2008; Keane 2013). To simplify the problem of characterizing heterogeneous wildland fuels, fuel description systems were developed to categorize fuels based on compilations of fuel attributes (Keane 2013). Resulting “fuel types” describe “identifiable *associations* of fuel elements with distinctive species, form, size, arrangement, and continuity that will exhibit characteristic fire behaviour under defined burning conditions” (Merrill and Alexander 1987). Multiple fuel description systems exist internationally (e.g., Canada, the USA, Europe, and Australia). Fuel types commonly serve as inputs for fire behavior prediction systems (Anderson 1982; Forestry Canada Fire Danger Group 1992), whereby fire behavior (e.g., rate of spread, flame length, intensity) relationships with weather and topography are developed for each fuel type.

Fuel typing systems often overlap in the fuel variables used to characterize live and dead vegetation complexes, which can be derived directly from field measurements or inferred from remotely sensed data (Keane 2013). Fuel types frequently divide fuelbeds into three layers: ground and surface fuels, which drive surface fire behavior; canopy or overstory fuels, which drive crown fire behavior; and understory, subcanopy, or ladder fuels, which mediate the transition from surface to crown fire (Table 1) (Keane et al. 2001; Keane 2015). Despite similarities in fuel attributes, fuel description systems often differ in their application (Sandberg et al. 2001; Scott and Burgan 2005; Keane 2013, 2015). For example, in *fuel association* approaches (as above), fuel information is assigned to categorical fuel types defined by the dominant vegetation species (Keane 2013; Phelps and Beverly 2022). In contrast, *fuel classification* approaches directly or indirectly cluster fuelbeds (the sum of live and dead flammable biomass, often organized by stratum) into unique groups based on fuel attributes (Keane 2013). Independent of the approach, some measure of fuel attributes is required to reliably characterize fuelbeds and classify fuel types.

**Table 1:** Fuel attributes commonly used to categorize fuel types and predict fire behavior

Layer/attribute	Units
**Canopy fuels**	
Canopy base height	m
Canopy stand height	m
Canopy fuel load and bulk density	kg m^−2^ and kg m^−3^
Canopy cover	%
Canopy composition	Species and proportion
**Understory and ladder fuels**	
Understory base height	m
Understory height	m
Understory fuel load and bulk density	kg m^−2^ and kg m^−3^
Understory composition	Species and proportion
Ladder fuel load	kg m ^−2^
Ladder fuel composition	Type and size
**Surface and ground fuels**	
Surface fuelbed height	m
Surface fuel load	kg m^−2^
Surface fuel composition	Type and size
Ground fuelbed depth	cm
Ground fuel load	kg m^−2^

In Canada, the Fire Behavior Prediction (FBP) System, a subsystem of the Canadian Forest Fire Danger Rating System (CFFDRS), uses a *fuel association* method to describe 16 categorial fuel types based primarily on structurally defined forest landcover classes (Table 2) (Forestry Canada Fire Danger Group 1992; Wotton et al. 2009). The FBP System was designed to predict stand-level fire rate of spread, fuel consumption, and fireline intensity to aid in fire suppression planning and field operations. In the FBP System, fuel types are assigned based on generalizable qualitative characteristics of the forest floor and organic layer, surface and ladder fuels, forest stand characteristics, and broadly defined characteristics of forest structure and composition. For example, the C-3 Mature Jack or Lodgepole Pine fuel type is characterized by dense (1000–2000 stems/ha) mature jack pine (*Pinus banksiana* Lamb.) or lodgepole pine (*Pinus contorta* Dougl. ex Loud.), with complete crown closure, 8-m crown base height, and light understory and surface fuels over a moderately deep (10 cm) and compacted feather moss (*Pleurozium schreberi* (Willd. ex Brid.) Mitt.) layer (Table 2, Forestry Canada Fire Danger Group 1992). For selected fuel types, the FBP System also considers seasonality, reflecting the phenological stage of the dominant vegetation (green up, grass curing), and the percentage conifer stand fraction (% C), which modifies the predicted rate of spread (Table 2, Forestry Canada Fire Danger Group 1992; Wotton et al. 2009; Alexander 2010).

**Table 2:** Fuel types described by the Canadian Forest Fire Behavior Prediction (FBP) System

Group/identifier	Descriptive name
**Coniferous**	
C-1	Spruce-lichen woodland
C-2	Boreal spruce
C-3	Mature jack or lodgepole pine
C-4	Immature jack or lodgepole pine
C-5	Red and white pine
C-6^a^	Coniferous plantation
C-7	Ponderosa pine or Douglas-fir
**Deciduous**	
D-1	Aspen–leafless
D-2^*^	Aspen–green
**Mixedwood**	
M-1^b^	Boreal mixedwood–leafless
M-2^b^	Boreal mixedwood–green
M-3^c^	Dead balsam fir mixedwood–leafless
M-4^c^	Dead balsam fir mixedwood–green
**Slash**	
S-1	Jack or lodgepole pine slash
S-2	White spruce, balsam slash
S-3	Coastal cedar, hemlock, Douglas-fir slash
**Open**	
O-1a/b^d^	Grass–matted or standing

^a^Can vary the crown base height

^b^Must specify percent conifer composition

^c^Must specify percent dead fir

^d^Must specify the degree of curing and can specify fuel load

^*^Unofficial (interim) fuel type (Alexander 2010)

Source: “Table 1 (List of fuel types presently included in the Canadian Forest Fire Behavior Prediction (FBP) System).” Updates and revisions to the 1992 Canadian Forest Fire Behavior Prediction System, B.M. Wotton, M.E. Alexander, S.W. Taylor, 2009. Canadian Forest Service, Natural Resources Canada. Reproduced with the permission of the Department of Natural Resources, 2023

The FBP System fuel types emphasize crown fire behavior in subboreal and boreal forests, with descriptions that mirror the forest inventory attributes routinely collected for forest harvesting (Phelps and Beverly 2022). However, variations in measured fuel characteristics within a single stand can be greater than variation between fuel types of adjacent stands (Brown and Bevins 1986; Miller et al. 2003). Thus, despite its convenience, this *fuel association* (Keane 2013) approach along with the limited number of FBP System fuel types often results in fuel characteristics and forest inventory attributes that deviate from FBP System fuel type descriptions (Phelps and Beverly 2022). As a result, the breadth and diversity of fuelbeds that cover British Columbia (BC), Canada, landscapes are inadequately represented by FBP System fuel types (Hawkes et al. 1995; Parisien et al. 2013; Perrakis et al. 2018). This lack of representation stems in-part from the sheer diversity of forests, fuels, and disturbance histories, which often differ from the characteristics of native subboreal and boreal forests for which the fuel types were developed. As a consequence, applications of the FBP System in interior BC require substantial subjective interpretation, because many fuelbeds are not described (Parisien et al. 2013; Perrakis et al. 2018).

In BC, spatial fuel type data are available from both provincial and national governments. Provincial fuel type maps are produced using an expert-informed decision tree (Perrakis et al. 2018), which associates vegetation attributes from provincial forest inventory data (Vegetation Resource Inventory [VRI], Ministry of Forests 2022) with FBP System fuel types. Across Canada, national fuel type maps are produced by the Canadian Forest Service (CFS) using a different, simplified decision tree process based on national forest inventory and landcover data (Beaudoin et al. 2014; Natural Resources Canada 2019). The provincial fuel type layer is widely used for wildfire field operations and fuel management, whereas the national fuel type layer is frequently used for broad-scale scientific research (e.g., Wotton et al. 2017; MacMillan et al. 2022; Coogan et al. 2022).

Despite widespread use, the accuracy and applicability of these fuel type maps to BC conditions have not been formally assessed through field validation. To address this knowledge and data gap, we assessed the applicability of FBP System fuel types to conditions in interior BC and quantified agreement between provincial and national fuel type maps and on-site assessments. We identified fuel typing mismatches—hereafter defined as differences in fuel typing arising from either misclassification, the lack of a representative fuel type, or both. We discuss the consequences of fuel type mismatches, identify challenges associated with needed but missing fuel types, and provide recommendations for improved fuel characterizations.

## Methods

### Study area

**Fig. 1:**
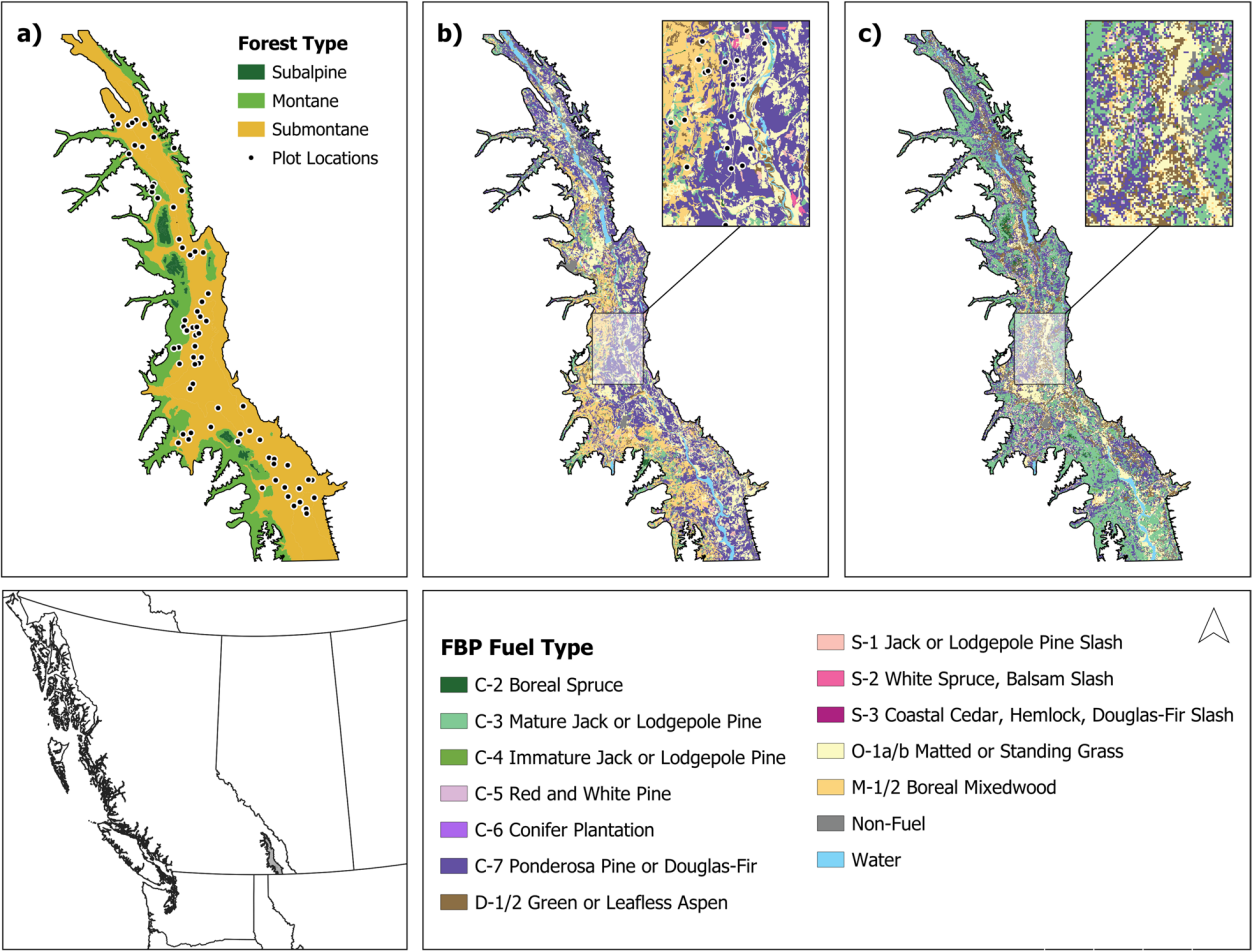
The southern Rocky Mountain Trench (RMT), located in southeastern British Columbia, Canada, showing **a** the distribution of forest types and location of field plots (*n* = 76), and Fire Behavior Prediction (FBP) System fuel typing for **b** 2020 provincial (British Columbia Wildfire Service; 50 m) and **c** 2019 national (Canadian Forest Service; 250 m) data

Our study area is the southern Rocky Mountain Trench (RMT) of southeastern BC, which separates the Columbia Mountains to the west from the Rocky Mountains to the east (Fig. 1). The RMT is comprised of hot submontane (750–1050 m, Interior Douglas-fir zone), warm montane (1050–1500 m, Montane Spruce zone), and cool subalpine (1500–2100 m, Engelmann Spruce-Subalpine Fir zone) ecosystems across an elevational gradient (MacKillop 2018). Hot and dry submontane ecosystems in the valley bottom are co-dominated by interior Douglas-fir (*Pseudotsuga menziesii* var. *glauca* (Mayr) Franco), ponderosa pine (*Pinus ponderosa* Douglas *ex* Lawson), and western larch (*Larix occidentalis* Nutt). These dry forest ecosystems historically consisted of highly varied patchworks of grasslands, woodlands, and open forests maintained by frequent low-intensity fires (Greene 2021). Warm and dry montane forests occur on steep slopes adjacent to the valley floor, consisting of interior Douglas-fir, lodgepole pine (*Pinus contorta* Douglas *ex* Louden), western larch, and hybrid spruce (*Picea engelmanni* Parry *ex* Engelm. x *P. glauca* (Moench) Voss). Historically, a moderate or mixed-severity fire regime dominated at these elevations (Marcoux et al. 2013, 2015). In the upper montane elevations, cool and dry subalpine forests of subalpine fir (*Abies lasiocarpa* (Hook.) Nutt.), Engelmann spruce (*Picea engelmanni*), and lodgepole pine forests dominate and are characterized by mixed- and high-severity fire regimes (Marcoux et al. 2013, 2015).

Over a century of fire exclusion and timber harvesting have altered stand size and age-class structure, increased stand density, and altered species composition across forest types (Marcoux et al. 2015; Hessburg et al. 2019; Greene 2021; Hagmann et al. 2021). Today, dense submontane forests infilled with immature and suppressed Douglas-fir in multi-layered arrangements are abundant. Drought and Douglas-fir beetle (*Dendroctonus pseudotsugae* Hopkins) have further stressed and degraded remaining mature interior Douglas-fir stands (MacKillop 2018; Greene 2021). Clear-cut and seed-tree regeneration harvest systems result in a patchwork of cutblocks of variable size and age. Replanting after harvesting has produced dense plantations of lodgepole pine seedlings, saplings, and poles in montane forests. Extremely large outbreaks of the mountain pine beetle (*Dendroctonus ponderosae* Hopkins) have occurred as a result of forest densification and increased connectivity of mature lodgepole pine forests in the absence of fire, combined with increasing annual and seasonal drought (Carroll et al. 2003; Taylor and Carroll 2003; Walton 2013).

### Plot selection

During May and June of 2022, we applied a stratified-random sampling design to establish 76 1-ha circular plots capturing a 200-km north–south gradient spanning the southern RMT (607,000 ha) (Fig. 1). To control for the impact of forest density, the study area was stratified by landcover class using tree canopy cover data from the provincial forest inventory (Ministry of Forests 2022) in a geographic information system (GIS). Classes included areas of grassland (0–5% tree canopy cover, *n* = 9), woodland (6–15% canopy cover, *n* = 11), open forest (16–35% canopy cover, *n* = 11), closed forest (36–59% canopy cover, *n* = 14), dense forest (≥ 60% canopy cover, *n* = 19), and recently harvested forest (calendar year of harvest ≥ 2000, *n* = 12) (Taylor et al. 1998). Within each class, we randomly selected plots located on public (crown) land. Plot locations were randomly placed 50 to 200 m from accessible (all weather, permanent) resource roads using a GIS. In the field, hand-held global positioning devices (positional accuracy ± 5 m) were used to locate plot centers.

### Fuel typing

We applied three approaches for classifying fuel types according to the Canadian FBP System. First, for each of the 76 plots, we collected fuel attribute data and assigned an FBP System fuel type in the field (*field assigned fuel type*). Second, to address discrepancies between the subjective application of the FBP System and the provincial decision tree, we used the field attributes to assign a fuel type through the provincial decision tree (*field decision tree fuel type*). Finally, using provincial and national remotely sensed fuel type maps, we extracted the respective assigned fuel type at each plot (*provincial and national fuel types*).

#### Field-assigned fuel type

In the field, we compared the structure and composition of each 1-ha circular plot to the photo references and FBP System supporting documentation (Forestry Canada Fire Danger Group 1992) to determine (a) whether a fuel type existed (Yes/No) within the FBP System to characterize the stand and (b) which fuel type best matched the observed stand structure and/or composition. *Field-assigned fuel types* were determined without knowledge of the provincial and national fuel type assignments. *Field-assigned fuel types* were based on the forest floor and organic layer, surface and ladder fuels, and stand structure and composition, as described by the FBP System (Table 2). To reduce the potential for unintentionally creating discrepancies between the timing of field assessments and the aseasonal remotely sensed data, we did not consider seasonal fuel type variants (prior to and following green up, grass curing). Similarly, for mixedwood stands (defined as 20 to 80% deciduous species, Perrakis et al. 2018), we did not consider the percent conifer attribute in our analysis, which further modifies predictions within the M-1/M-2 fuel types (Forestry Canada Fire Danger Group 1992; Wotton et al. 2009).

Fuel attribute data were collected and stored using ArcGIS Survey123 (see Additional file 1: Appendix A). At the plot center, we took photographs in the four cardinal directions and two vertical projections (up, down). Three additional photos were taken from the plot center to more fully represent canopy and elevated fuels, ladder and midstory fuels, and surface and ground fuels. To assign a fuel type in the field, we used the forest inventory attributes recorded in the field decision tree (see the section “Field decision tree fuel type”) and added measurements of the forest and organic layer, surface fuels (composition, amount, and height), ladder fuels (composition, amount, height, and continuity), and any evidence of live vegetation stress.

A common challenge in the assignment of an FBP System fuel type is that it requires subjective interpretation (Hawkes et al. 1995; Perrakis et al. 2018). We minimized these subjective aspects of the system through technical training, documentation, and consultation with FBP System experts and the broader community of practice. In cases where there was no suitable FBP System fuel type to characterize a plot, we assigned a fuel type based on the closest approximation to structure and composition through consultations and guidance from FBP System experts (BC Wildfire Service, personal communication; D. Perrakis and S. Taylor, Canadian Forest Service, personal communication).

#### Field decision tree fuel type

The provincial decision tree process considers 21 attributes of forest inventory polygon data to assign fuel types (Perrakis et al. 2018). Attributes include the land cover type, biogeoclimatic zone and subzone, year of the most recent harvesting, year and type of the earliest non-harvesting disturbance, canopy cover (%), density of live and dead overstory trees (stems/ha), percentage of dead overstory trees (%), species codes and percentages of the two most dominant tree species, canopy tree height (m) and age (years) of the leading species (Ministry of Forests, Lands and Natural Resource Operations 2016; Ministry of Forests, Lands and Natural Resource Operations 2019). Fuel types are then assigned based on canopy species, stand structure, and assumed relationships between stand structure characteristics and fire behavior. In their report, the developers of the provincial fuel typing decision tree noted that their fuel type maps could be significantly improved by field validation of vegetation and fuel structure (Perrakis et al. 2018). Thus, to address discrepancies between the subjective application of the FBP System and the provincial decision tree, we processed the field attributes through the provincial decision tree to produce the *field decision tree fuel type*.

At each 1-ha plot, we collected the field attributes required to assign a fuel type through the provincial decision tree. Landcover was assigned in the field using four customary variables that describe a plot as vegetated or non-vegetated, treed or non-treed, site location relative to elevation and drainage (alpine, wetland, or upland), and density based on canopy cover (dense 61–100%, open 26–60%, sparse 10–25%). Biogeoclimatic zone and subzone were assigned in a GIS using the most current (2021) provincial classification (Province of British Columbia 2022). To assess harvesting and non-harvesting disturbances, we recorded disturbance type in the field, differentiating between harvesting (clear-cut, selective, seed-tree), mechanical thinning and/or pruning, wildfire, prescribed fire, windthrow, and insect outbreaks. Time since recent disturbance (≤ 10 years) was also estimated in the field and, in the case of harvesting, confirmed in a GIS with provincial forest inventory data. Canopy cover was measured to the nearest 5% (0–100% scale) along a 40-m transect using a GRS densitometer, where each observation along the transect was classified as conifer, broadleaf, mixed, or no cover at 2-m intervals.

To sample live and dead subcanopy trees (≤ 7.5-cm diameter at breast height [DBH]), we created a variable radius plot of 5.64 m for open stands and 3.99 m for dense stands. Plot radii were established to obtain a count of at least 20 trees per plot. To sample live and dead canopy trees, we created a variable radius plot of 11.28 m for open stands and 7.98 m for dense stands, with plot radii again selected to obtain a count of ≥ 20 trees per plot. Canopy density measures were recorded separately for live and dead stems for size classes 7.5–12.49 cm DBH and ≥ 12.5 cm DBH. To derive species percentages, we measured canopy basal area using a BAF 2 or 3 prism to obtain a count of 8–10 trees per plot and recorded live trees by species in order of dominance. To measure stand-level canopy height and age at each plot, we first identified a representative dominant canopy tree and then used an increment borer to assign age in years and a clinometer to measure tree height to the nearest 0.5 m.

To derive *field decision tree fuel types*, we coded the Perrakis et al. (2018) decision tree into a Python (Python Software Foundation 2023) script (BC Wildfire Fuel Typing, bcwft)and used it to post-process the field attributes by assigning a fuel type and fuel typing process number linked to the decision criteria at each plot (Greene 2023, Table S1, Table S2). Ongoing internal reviews to the provincial decision tree since 2018 (Perrakis et al. 2018) have resulted in a revision to select branches related to the processing of disturbances (BCWS, personal communications), but to our knowledge did not influence fuel types at the sampled plots.

#### Provincial and national fuel types

To assess the *provincial-scale fuel typing* data, we acquired the map and database of fuel types from the BC Wildfire Service (BC Wildfire Service 2022) and extracted the fuel type assigned to each plot in a GIS (Fig. 1). In BC, forest inventory (VRI) attributes are derived from manual interpretation of aerial photographs, with annual updates from projected growth and yield modeling and disturbance data identifying harvesting, wildfire, and insect disturbances (Ministry of Forests 2022). The map of provincial fuel types is produced by applying the decision tree (Perrakis et al. 2018) to forest inventory attributes (Ministry of Forests 2022) and assigning a fuel type to each inventory polygon. Provincial fuel typing data also includes an attribute describing fuel typing confidence (low, medium, medium/high, high), which we extracted for each plot. We used the most recent vector version of this dataset, rasterized to 50-m resolution in a GIS.

To assess a *national-scale fuel typing* product, we acquired the national FBP System fuel type layer from the Canadian Forest Service (CFS, Natural Resources Canada 2019) and extracted the fuel type assigned to each plot in a GIS (Fig. 1). This fuel data layer consists of a 250-m raster map developed from remotely sensed national forest inventory and landcover data sets. The national process applies a decision tree to attributes including landcover classification, ecoregion, canopy cover, canopy height, and species composition to assign FBP System fuel types at a national level (Beaudoin et al. 2014; Natural Resources Canada 2019). The national fuel typing layer  is not designed for operational wildland fire management due to its lower resolution and limited availability of input attributes, but is often used for broad-scale research and national-level assessments.

### Data analysis

To assess the suitability of the 16 FBP System fuel types for characterizing fuels across the study domain, we summarized the number of plots that partially matched an FBP System fuel type in terms of structure and/or composition, versus those where no partially representative fuel type was available. We quantified the level of agreement or mismatch between fuel typing data layers with confusion matrices using the caret package in R (Kuhn 2022; R Core Team 2023). To explore discrepancies between field interpretations of fuels and existing fuel typing data, we conducted comparisons between field assigned, and provincial and national data (hereafter, *field observed agreement*). To explore the influence of forest inventory accuracy on fuel type, we held the decision tree process constant and conducted comparisons between provincial and field decision tree data (hereafter, *forest inventory agreement*). To explore the influence of the decision tree process without the influence of forest inventory data, we conducted comparisons between field decision tree and field assigned data (hereafter, *field decision tree agreement*). Finally, independent of field data, we conducted comparisons between provincial and national data (hereafter, *scaling agreement*). We then assessed and interpreted the specific levels of agreement and reasons for mismatches in fuel typing between the data sources.

To quantify the agreement between provincial forest inventory and field measurements of attributes used in the decision tree process, we extracted leading species, landcover class (vegetation density), canopy cover, canopy height, and live tree density from the provincial forest inventory (Ministry of Forests 2022) at plot locations in a GIS. We quantified the agreement between forest inventory and field measurements of attributes using confusion matrices for categorical variables and paired one-sided *t*-tests for continuous variables in R (R Core Team 2023).

## Results

### Fuel typing suitability

**Fig. 2:**
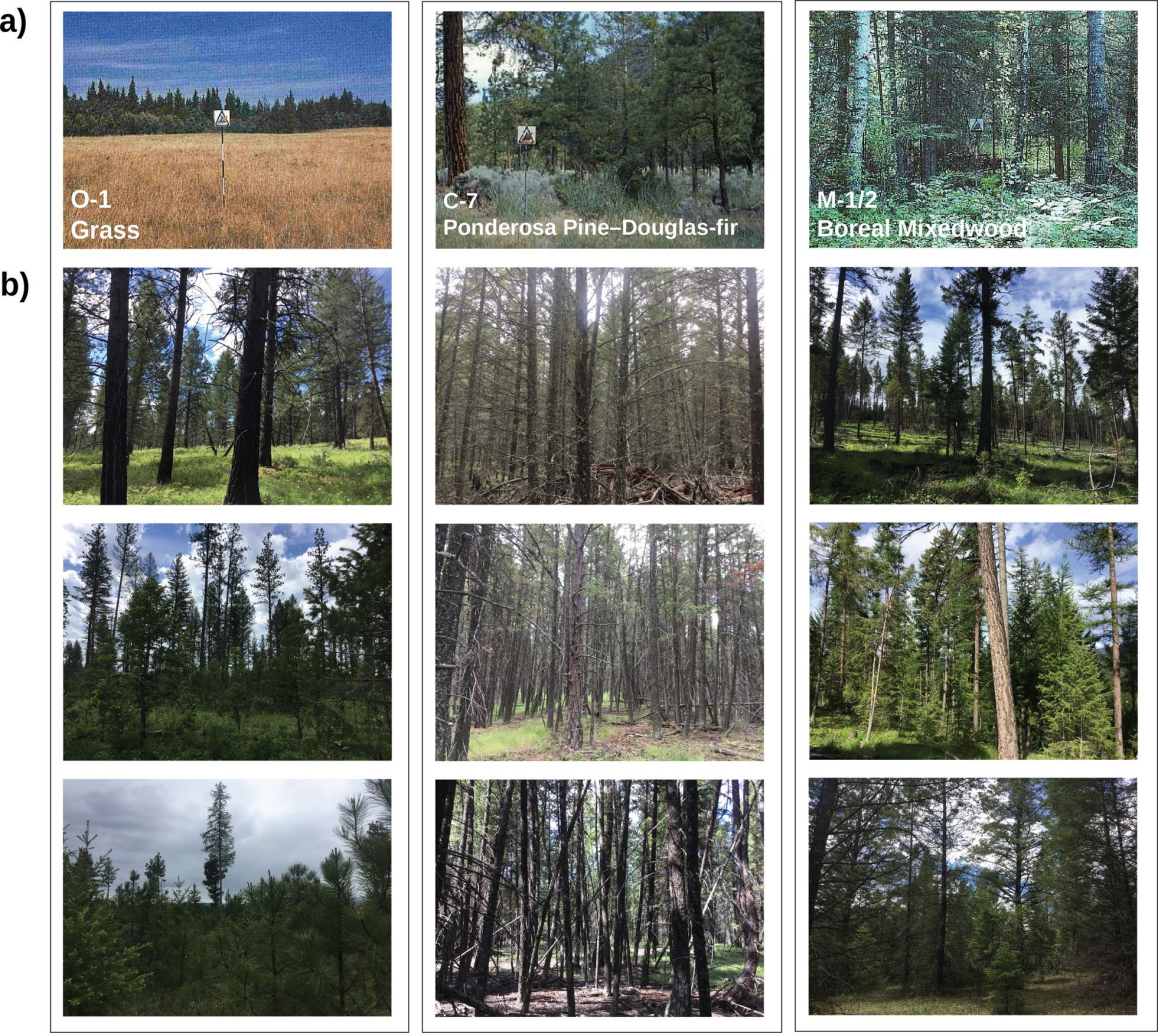
**a** Reference images from the Canadian Forest Fire Danger Rating System (CFFDRS) of Fire Behavior Prediction (FBP) System fuel types. Source: “FBP Fuel Type Description Webpage (https://cwfis.cfs.nrcan.gc.ca/background/fueltypes/c1).” Canadian Forest Service, Natural Resources Canada. Reproduced with the permission of the Department of Natural Resources, 2023. **b** Images of stand structures with recurring mismatches between field assigned and provincial fuel typing. Provincial fuel typing classified these stands as O-1 matted or standing grass, C-7 ponderosa pine or Douglas-fir, or M-1/2 boreal mixedwood, respectively

**Fig. 3:**
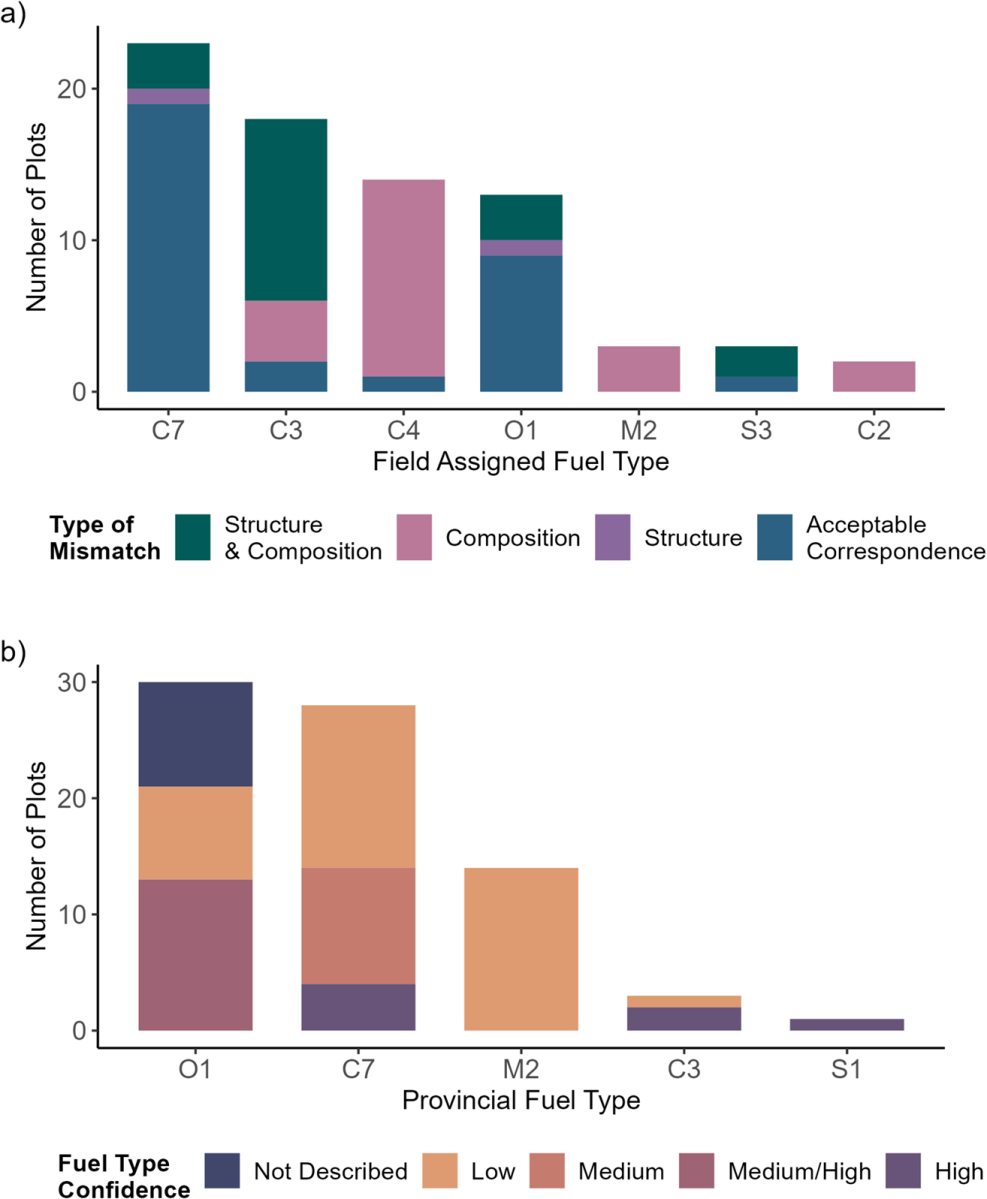
**a** Applicability of FBP System fuel types to field plots in interior British Columbia for field assigned fuel types. Applicability is scored as being mismatched to both structure and composition (26.3%, *n* = 20), mismatching composition (28.9%, *n* = 22), mismatching structure (2.6%, *n* = 2), or acceptable correspondence (42.1%, *n* = 32). **b** Fuel type confidence, assigned by BCWS, scored as low (48.7%, *n* = 37), medium (13.2%, *n* = 10), medium/high (17.1%, *n* = 13), high (9.2%, *n* = 7), or not described (11.8%, *n* = 9) for provincial fuel types

The most common fuel types assigned in the field were C-7 (Ponderosa Pine–Douglas-Fir, *n* = 23), C-3 (Mature Jack or Lodgepole Pine, *n* = 18), C-4 (Immature Jack or Lodgepole Pine, *n* = 14), and O-1 (Grass, *n* = 13) (Fig. 2) (Table 3). Of the 76 field plots assessed, we found that 42.1% of plots (*n* = 32) could be assigned a partially suitable FBP System fuel type in the field (Fig. 3). The remaining 57.9% of plots (*n* = 44) could not be assigned a partially suitable fuel type within the FBP System with respect to fuel structure and composition. Of these plots, 28.9% (*n* = 22) matched an FBP System fuel type in structure but not composition, 2.6% (*n* = 2) matched an FBP System fuel type in composition but not structure, and 26.3% (*n* = 20) did not match an FBP System fuel type in either structure or composition (Fig. 3). Fuel types used to classify these plots include C-3 (*n* = 16) and C-4 (*n* = 13), C-7 (*n* = 4), O-1 (*n* = 4), M-2 (*n* = 3), C-2 (*n* = 2), and S-3 (*n* = 2) (Fig. 3).

**Table 3:** Frequency of FBP System fuel type assignment to field plots (*n* = 76) for field assigned, field decision tree, provincial, and national approaches

	**Fuel typing approach**			
**FBP fuel type**	Field assigned	Field decision tree	Provincial	National
C-2	2	-	-	2
C-3	18	8	3	14
C-4	14	-	-	-
C-5	-	-	-	2
C-7	23	47	28	22
D-2	-	6	-	8
M-2	3	8	14	3
S-1	-	2	1	-
S-3	3	-	-	-
O-1	13	5	30	22
NF	-	-	-	3

See Table 2 for the description of fuel types

*NF* non-fuel

In the provincial fuel typing layer, the province describes confidence in their assignment of fuel type as low for 48.7% of plots (*n* = 37), medium for 13.2% of plots (*n* = 10), medium/high for 17.1% of plots (*n* = 13), high for 9.2% of plots (*n* = 7), and did not list a confidence level for 11.8% of plots (*n* = 9) (Fig. 3). Province-assigned confidence in fuel typing was low for C-7 (*n* = 14), M-2 (*n* = 14), O-1 (*n* = 8), and C-3 (*n* = 1), medium for C-7 (*n* = 10), medium/high for O-1 (*n* = 13), and high for C-7 (*n* = 4), C-3 (*n* = 2), and S-1 (*n* = 1). Fuel type confidence was not assigned by BCWS for 9 plots classified as O-1 (Fig. 3).

### Fuel typing comparisons

**Fig. 4:**
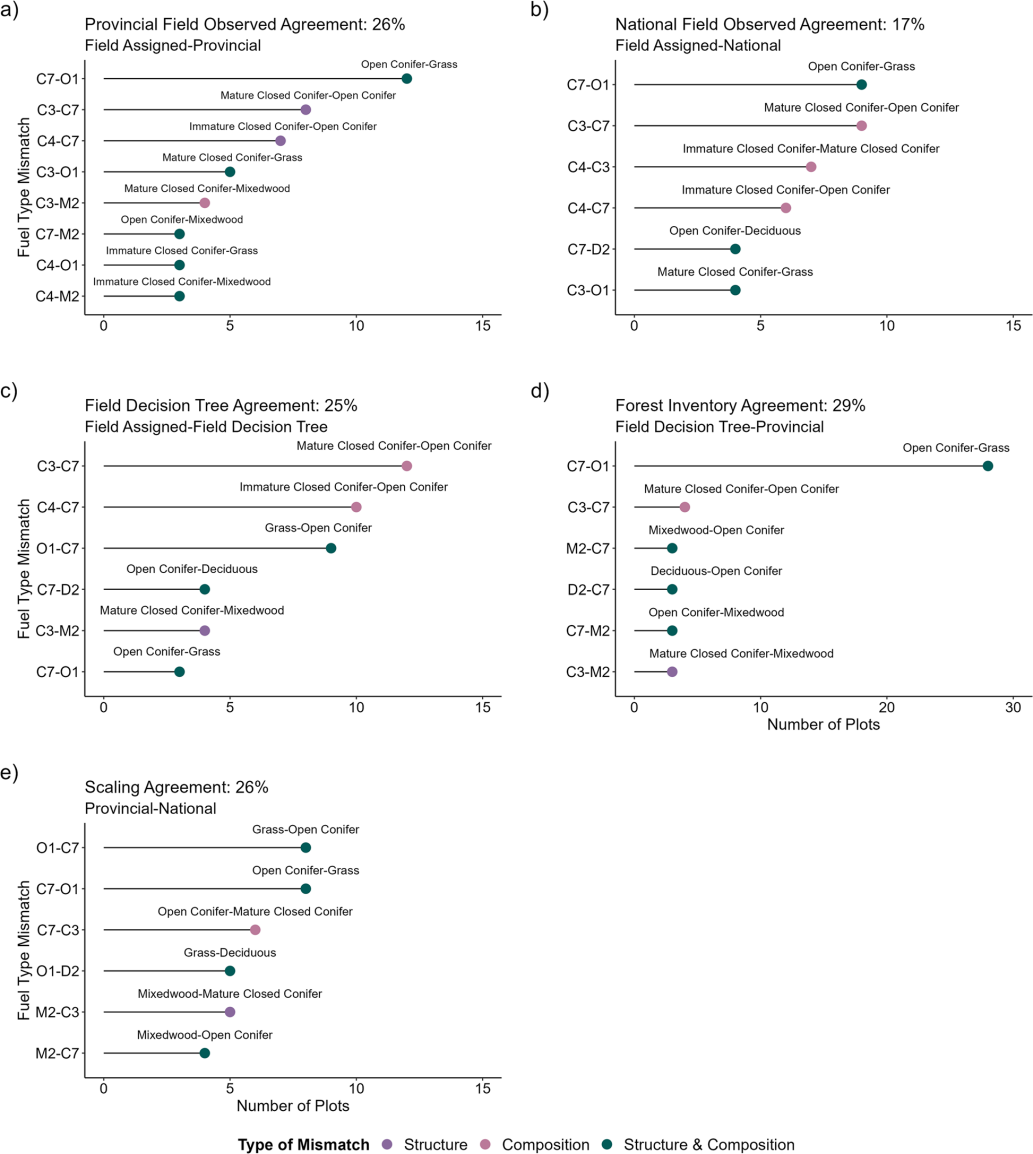
Frequent (≥ 3 plots) fuel typing mismatches for** a** provincial field observed agreement (provincial-field assigned), **b** national field observed agreement (national-field assigned), **c** field decision tree agreement (field decision tree-field assigned), **d** forest inventory agreement (provincial-field decision tree), and **e** scaling agreement (national-provincial). Labels reflect generalized descriptions of fuel types (e.g., C-3 Mature Jack or Lodgepole Pine is labeled as Mature Closed Conifer). Type of mismatch describes whether the fuel types differ in structure, composition, or structure and composition

We found consistently low *observed agreement* between fuel types assigned in the field and fuel types derived from provincial and national maps. Specifically, comparing field assigned and provincial fuel types, we found *provincial field observed agreement* at 26% of plots (*n* = 20) (Fig. 2, Table 4). Frequent mismatches (field assigned to provincial) occurred between C-7 and O-1 (*n* = 12), C-3 and C-7 (*n* = 8), and C-4 and C-7 (*n* = 7) (Fig. 2, Table 4). Comparing field assigned and national fuel types, we found *national field observed agreement* at 17% of plots (*n* = 13) (Table 4). Frequent mismatches (field assigned to national) occurred between C-7 and O-1 (*n* = 9), C-3 and C- 7 (*n* = 9), C-4 and C-3 (*n* = 7), and C-4 and C-7 (*n* = 6) (Fig. 4).

**Table 4:** Frequency of fuel typing mismatches (number of plots, % of plots) overall and by FBP System fuel type for provincial field observed disagreement (provincial-field assigned), national field observation disagreement (national-field assigned), field decision tree disagreement (field decision tree-field assigned), forest inventory disagreement (provincial-field decision tree), and scaling disagreement (national-provincial). Fuel types are presented in terms of the predicted fuel type (e.g., provincial) as compared to the reference (e.g., field assigned). Frequent mismatches are shown in Fig. 4

	Field observed disagreement	Field observed disagreement	Field decision tree disagreement	Forest inventory disagreement	Scaling disagreement
**FBP fuel type**	*Provincial*	*National*	*Field decision tree*	*Provincial*	*National*
C-2	-	2 (100%)	-	-	2 (100%)
C-3	2 (67%)	11 (79%)	6 (75%)	2 (67%)	13 (93%)
C-4	-	-	-	-	-
C-5	-	2 (100%)	-	-	2 (100%)
C-7	20 (71%)	19 (86%)	33 (70%)	13 (46%)	14 (64%)
D-2	-	8 (100%)	6 (100%)	-	8 (100%)
M-2	12 (86%)	3 (100%)	7 (88%)	9 (64%)	3 (100%)
S-1	1 (100%)	-	2 (100%)	1 (100%)	-
S-3	-	-	-	-	-
O-1	21 (70%)	15 (68%)	3 (60%)	29 (97%)	11 (50%)
NF	-	3 (100%)	-	-	3 (100%)
**Overall**	56 (74%)	63 (83%)	57 (75%)	54 (71%)	56 (74%)

See Table 2 for the description of fuel types

*NF* non-fuel

We also found low agreement when isolating the decision tree process (*field decision tree agreement*, holding data constant) and the forest inventory data (*forest inventory agreement*, holding process constant). Comparing field assigned and field decision tree fuel types, we found *field decision tree agreement* at 25% of plots (*n* = 19) (Table 4). Frequent mismatches (field assigned to field decision tree), occurred between C-3 and C-7 (*n* = 12), C-4 and C-7 (*n* = 10), and O-1 and C-7 (*n* = 9) (Fig. 4). Comparing field decision tree and provincial fuel types, we found *forest inventory agreement* at 29% of plots (*n* = 22) (Table 4). Frequent mismatches (field decision tree to provincial) occurred between C-7 and O-1 (*n* = 28) (Fig. 4). Finally, we found low agreement when comparing provincial (50 m) and national (250 m) remotely sensed approaches at different resolutions. Specifically, we found *scaling agreement* at 26% of plots (*n* = 20) (Table 4). Frequent mismatches (provincial-national) occurred between O-1 and C-7 (*n* = 8), C-7 and O-1 (*n* = 8), and C-7 and C-3 (*n* = 6) (Fig. 4).

### Forest inventory attributes

**Fig. 5:**
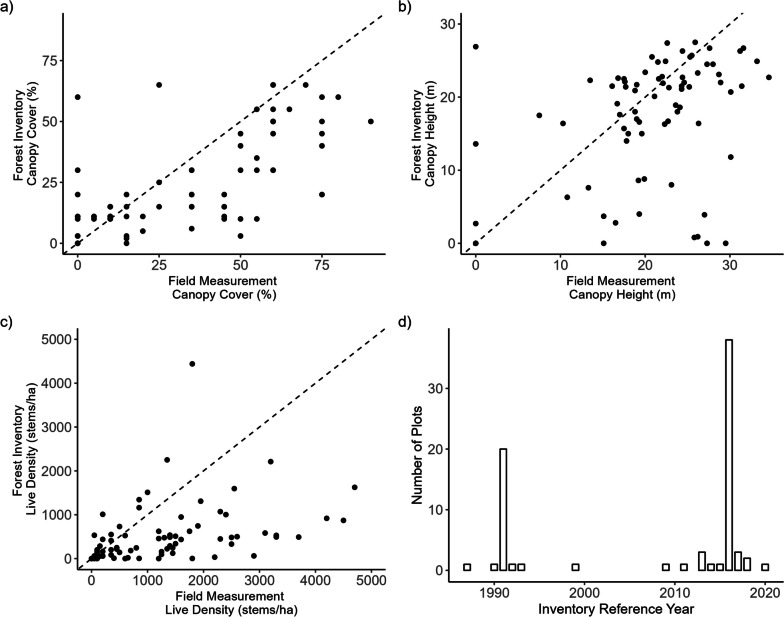
Agreement between photo-interpreted forest inventory attributes and field measurements of **a** canopy cover (%), **b** canopy height (m), and **c** live density (stems/ha). Dashed lines plot a 1:1 relationship between variables. On average, forest inventory data underpredicts canopy cover by 9.4%, underpredicts canopy height by 3.6 m, and underpredicts live density by 765 stems/ha, relative to field measurements. **d** Inventory reference year for photo-interpreted forest inventory data

Comparing provincial forest inventory (VRI) and field attributes, we found agreement of 58% (*n* = 44) for the first leading species, with frequent mismatches (field-forest inventory) between ponderosa pine and Douglas-fir (*n* = 10), and Douglas-fir and western larch (*n* = 5). Agreement for landcover class (vegetation density: spare, open, dense) was 45% (*n* = 34), with frequent mismatches (field-forest inventory) between open and sparse (*n* = 11), no class (< 10% cover) and sparse (*n* = 8), and dense and open (*n* = 7). Relative to field measurements, forest inventory data underpredicted canopy cover by 9.4% (SD = 19.7%, *p* < 0.0001, *t*_(75)_ = 4.2), canopy height by 3.6 m (SD = 9.2 m, *p* < 0.001, *t*_(75)_ = 3.4), and live density by 765 stems/ha (SD = 1102 stems/ha, *p* < 0.0001; *t*_(75)_ = 6.1) (Fig. 5).

## Discussion

### Fuel typing mismatches

We found consistently low agreement for provincial and national fuel typing, relative to field-based classification. Below we describe fuel typing challenges for (i) dry interior ecosystems, (ii) mixedwood and deciduous fuel types, and (iii) post-harvesting conditions in interior BC. Additional forest conditions that present fuel typing challenges, including stands influenced by mountain pine beetle outbreaks, hybrid spruce forests, mesic montane valleys, and coastal rainforests, have been identified elsewhere but were not sampled in this study (Hawkes et al. 1995; Perrakis et al. 2014, 2018).

#### Mismatch: dry interior ecosystems

In dry interior ecosystems, we encountered widely varied fuelbeds and forest structural conditions influenced by a history of natural disturbances and management. Mismatches for both provincial and national fuel types (*field observed agreement* between O-1 and C-7, C-7 and C-3, C-7 and C-4, C-3 and C-4) reveal discrepancies across gradients of size and age class, canopy cover, canopy and subcanopy density, and surface fuel composition and loading conditions. These conditions result from a shared history of land use, fire suppression, and industrial forest management in historically fire-adapted ecosystems with active fire regimes (Hessburg et al. 2019; Greene 2021; Hagmann et al. 2021; Baron et al. 2022). However, there is no fuel type within the FBP System to describe the majority of these stand structures and compositions. Likewise, there is little opportunity within existing frameworks to modify or combine existing fuel types to address these mismatches.

In both provincial and national decision trees and fuel typing layers (Perrakis et al. 2018; Natural Resources Canada 2019), mismatches were frequently associated with branches of the decision tree that assign fuel type based on species composition (e.g., Douglas-fir as the leading overstory canopy species). This compositional approach limits the inclusion of structural fuels attributes, which are emphasized over composition in the FBP System and are important for predicting the likelihood of short or long flame lengths, active or passive tree torching, and crown fire initiation and spread (Cruz et al. 2005; Alexander and Cruz 2006, 2011; Cruz and Alexander 2013; Perrakis et al. 2023). For example, in the provincial decision tree, eight dry-zone Douglas-fir stands that had not been logged in the past 6 years, with canopy heights over 12 m and canopy cover greater than 55%, were assigned as C-7 based on composition (Perrakis et al. 2018), whereas the field assigned fuel type was C-4 based on structure (Table S1). The assignment of the open C-7 fuel type to these dense stands deviates from the structural descriptions of the FBP System and may underpredict potential fire behavior.

The C-7 fuel type was developed to represent stands frequently disturbed by low-severity surface fire, where multi-cohort regeneration for ponderosa pine and Douglas-fir in pure or mixed compositions is ongoing. These dynamics result in open forests with 10–50% canopy closure, light surface fuel accumulations, and shallow duff and litter layers (Forestry Canada Fire Danger Group 1992; Hagmann et al. 2021). However, due to the long absence of Indigenous cultural fires and the suppression of natural ignitions, the majority of dry forests in southeastern BC are in fire deficit, with between 6 and 10 fires missed (Baron et al. 2022). Open forests matching the C-7 fuel type exist almost exclusively in small, isolated ecological restoration treatments where fire has been re-introduced through prescribed burning (Greene 2021). The remaining fire-excluded forests have been infilled with immature, suppressed trees in multi-layered arrangements, altering historical patchworks at a landscape scale (Hessburg et al. 2019; Hagmann et al. 2021). These altered structural conditions are especially challenging to characterize from a surface and canopy fuel perspective due to the absence of field sampled data, and are likely being misrepresented by the C-7 fuel type.

#### Mismatch: mixedwood and deciduous forests

We also identified significant challenges in differentiating pure and mixed deciduous conifer (western larch) and broadleaf fuel compositions, evidenced by mismatches between mixedwood and conifer (M-2 and C-7, M-2 and C-3, D-2 and C-7) fuel types. The focus of concern here is that in the provincial and national decision trees, western larch is grouped with deciduous broadleaf trees such as trembling aspen (*Populus tremuloides* Michx.), based on the assumption that western larch cannot support crown fire and contributes to the broadleaf portion of the stand (Perrakis et al. 2018; Natural Resources Canada 2019). Pure western larch stands are assigned the D-1/D-2 fuel type, while mixed-conifer stands including western larch are assigned the M-1/M-2 fuel type. For example, using the provincial decision tree, two stands of mixed Douglas-fir and western larch that had not been logged in the past 6 years were assigned M-2 based on composition (Perrakis et al. 2018), whereas the field assigned fuel type was C-3 based on structure and composition (Table S1).

Although western larch and trembling aspen are both deciduous, they are markedly different in their adaptations to fire. Western larch is highly adapted to the presence of fire and frequent burning, as evidenced by the species’ thick bark, foliar geometry, relatively low resin content, tendency for lower branch pruning, and the deciduous nature of foliage (Schmid and Shearer 1995). Reports of fire behavior and post-fire mortality in pure and mixed western larch stands reveal that this species can persist under the influences of low- and moderate-severity fire (Hood et al. 2007; Hopkins et al. 2014; Marcoux et al. 2015). This stands in marked contrast to trembling aspens’ adaptations to fire. Aspen has a thin bark, is fast growing from seed and/or a clonal root system, and resprouts quickly to occupy sites as an early seral species after partial or complete stand-replacing fire (Baker 1925; Jones and DeByle 1985; Bradley et al. 1992). Moreover, aspen often limits fire growth and severity outcomes when in full leaf, but is more readily burned when leafless in the spring and fall (Perala 1974; Hély et al. 2000, 2001; Alexander 2010; Nesbit et al. 2023). In short, western larch is not equivalent to trembling aspen in predicted fire behavior. Based on our findings, the current extent of the M-1/M-2 and D-1/D-2 fuel types in provincial and national fuel type layers does not accurately represent the extent of these fuel conditions in interior forests.

#### Mismatch: post-harvesting conditions

Modern harvesting, slash disposal, and forest regeneration were poorly represented by the FBP System fuel types, resulting in frequent mismatches. Of the plots identified in the field as harvested (*n* = 26; clear-cut, seed-tree, or mechanical thinning), fuel typing mismatches occurred in 73% of provincial fuel types and 92% of national fuel types. Fuel structures after harvesting differ in the residual surface, ladder, and canopy fuels prior to slash treatment (Graham et al. 1999). In the provincial decision tree, all recently harvested stands are treated as clear-cuts and are assigned slash fuel types during the first 5 to 10 years after harvesting, based on the estimated time of harvesting and replanting (Perrakis et al. 2018).

The treatment of modern post-harvesting residue, and its influence on fire behavior, is poorly represented by the FBP System fuel types as surface fuelbeds. The FBP System slash fuel types are based on silvicultural practices from the 1970s and were developed from experimental burns where slash was scattered across cutblocks with fixed fuel load values (Forestry Canada Fire Danger Group 1992). In the 1970s and 1980s, broadcast burning was frequently used in BC to remove harvesting residues and pre-treat units for replanting; however, this practice dramatically declined after the 1990s (Hoffman et al. 2022) and was replaced with pile burning at landings and, less frequently, mastication of remaining fuels. New residue treatment methods, such as surface fuel mastication, are not described by the FBP System and are not well understood from a fire behavior perspective (Kreye et al. 2014). Surface fuel mastication can produce lower spread rates and intensities than traditional slash treatments, but can also result in prolonged flaming and smoldering combustion (Kreye et al. 2014; Schiks et al. 2015; Thompson et al. 2016). In addition, where slash deposits are initially quite high and where significant non-merchantable fuel ladders remain, fuel mastication results in fuel concentration and relocation to the forest floor rather than overall fuel reduction (Kreye et al. 2014; Prichard et al. 2021).

Sampling in young plantation forests also produced frequent mismatches in fuel typing. Young forest conditions, which occur nominally after the onset of regeneration but before crown closure (4–12-m height), can exhibit a range of fuel conditions that can be influential to fire behavior depending on the species composition, planting density, and spatial arrangement (North et al. 2019). In addition to the role of the regenerating overstory, fire behavior is heavily influenced by remnant surface fuels left behind after harvesting (Graham et al. 1999, 2004). Young plantations can exhibit higher fire severity and tree mortality than adjacent conifer forests, especially in the absence of surface fuel treatments after harvesting (Thompson et al. 2007; Lyons-Tinsley and Peterson 2012; Zald and Dunn 2018; North et al. 2019; Prichard et al. 2021). The C-6 (Conifer Plantation) fuel type in the FBP System initially appears pertinent to young plantations, but assumes complete canopy closure, no understory or shrub layer, and a continuous surface fuelbed of pine needle litter, with low predicted crown fire behavior relative to other forested fuel types (Forestry Canada Fire Danger Group 1992). Applications of this FBP System fuel type overlook the widespread variation in plantation composition, density, and surface fuelbed composition in BC. The C-6 fuel type is therefore inconsistent with the observed fuel structures and fire behavior occurring in modern-day plantations and is infrequently applied during fire suppression operations for this reason (Perrakis et al. 2018; BCWS, personal communications), leaving a gap in the representation of young forest conditions.

### Sources of mismatches

We identified two main sources of fuel typing mismatches: (i) the accuracy and limited availability of forest inventory data and (ii) the suitability of the fuel typing system to represent conditions encountered in the field and across spatial scales.

#### Source of mismatches: forest inventory data

Through assessments of *forest inventory agreement*, we identified the accuracy and availability of forest inventory data as a critical factor underpinning fuel typing mismatches. We found that on average forest inventory data underpredicted canopy cover by 9.4%, canopy height by 3.6 m, and live density by 765 stems/ha, relative to field measurements. Mismatches that result from applying the decision tree to forest inventory data are a direct consequence of inconsistencies in interpreting attributes from aerial photography without field validation (Bourgeois et al. 2018; Tompalski et al. 2021). Due to its reliance on photo interpretation, provincial forest inventory data is derived from imagery that is frequently outdated at local scales (Ministry of Forests 2019). For example, for 33% of plots considered in this study (*n* = 25), the most recent reference imagery pre-dates the year 2000. Inconsistent aerial photographic acquisition dates produce widespread propagation of error when attributes are projected to present. Time lags for incorporating updates, and the cumulative effects of ongoing dynamic natural and human disturbances, further compound errors.

Fuels characterizations across BC are limited to the available attributes in the provincial forest inventory (Ministry of Forests 2022). The provincial forest inventory was originally designed to inventory and account for merchantable timber, but does not consistently account for non-merchantable timber conditions. Reliance of the forest inventory on air photo interpretation with limited field validation further biases the quantification of canopy conditions, because there is limited ability to observe critical ladder and surface fuels (Arroyo et al. 2008; Gale et al. 2021). The persistent underestimation of the density of live trees in our study is likely a result of inconsistent image acquisition year, concealment of understory conditions by an overhead canopy, and a bias toward inventorying merchantable timber. Of the 18 attributes identified as relevant to represent fuels and predict fire behavior (Table 1), only three exist in provincial forest inventory data: canopy height, canopy cover, and canopy species and proportion. Notably, critical surface and canopy fuel attributes including canopy base height, canopy bulk density, and surface fuel load are not inventoried and cannot be accurately derived with existing data. These results suggest that photo-interpreted forest inventory approaches struggle to accurately characterize structural fuel attributes, limiting application for fuel characterizations.

#### Source of mismatches: fuel typing system

Although fuel typing is currently limited by the availability of suitable fuel data, the broader applicability and spatial scaling of the FBP System fundamentally underpins fuel typing mismatches. The FBP System fuel types were formally developed in the 1970s and 1980s through an experimental burning program (Lawson et al. 1985; Van Wagner 1989) to predict individual, stand-level fire spread and behavior to aid fire suppression (Forestry Canada Fire Danger Group 1992). The 16 forest fuel types reflect the amount of empirical fire behavior data available in Canada at the time of development (over 400 fire observations as of 1989), but were never intended to be comprehensive or without revision (Stocks et al. 1989; Hawkes et al. 1995). When applied for its original purpose in boreal forests, under high and extreme fire weather conditions, the FBP System generally performs well. Although it was anticipated that additional fuel types would be later incorporated (Stocks et al. 1989), increasing difficulty in implementing experimental burning projects and changing government priorities limited FBP System advancements. A resulting challenge is that applying the FBP System requires substantial subjective interpretation, which is often described as a “blend of art and science” (Hawkes et al. 1995; Perrakis et al. 2018). Today, the FBP System is applied in fuel structures that it was not developed for, introducing multiple sources of error which propagate to challenge interpretations.

Scaling across spatial extents and resolutions further limits applications of the FBP System, which was designed using homogenous stand-level measurements and was not intended for heterogeneous landscape-level assessments (Forestry Canada Fire Danger Group 1992). Although spatialized fuel data is in high demand, efforts to spatialize the FBP System have repeatedly produced challenges related to missing fuel types and fuelbed heterogeneity (Hawkes et al. 1995; Perrakis et al. 2018). In this study, we identified low *scaling agreement* (26%) between provincial (50 m) and national (250 m) remote sensing approaches, arising from different forest inventory data at different resolutions challenging the implicit assumption of homogenous pixels. Fuels are highly variable across time and space (Keane et al. 2001), and within-stand fuel variability can equal or exceed variability between stands (Brown and Bevins 1986; Miller et al. 2003; Keane 2013). In the field, fuel characteristics at fine- to meso-scales are rarely correlated with forest inventory attributes (e.g., species, canopy height), because these variables vary across coarser scales than fuels—often arising from broad landscape disturbances and environmental gradients (Keane et al. 2012; Keane 2013). As a consequence, a single fuel type in the field may be a placeholder for many distinct fuelbeds.

### Implications of mismatches

We found that fuel types poorly match existing fuel conditions, introducing significant uncertainty and challenging operational applications. These mismatches make it difficult for fire managers to accurately determine expected fire behavior before an event occurs (BCWS, personal communications). Misrepresentations of fuels can decrease confidence in system predictions during suppression actions and impact operational decision-making (Crowley et al. 2023). For example, overpredicting fire behavior can lead to misapplied planned ignitions during suppression actions. Alternatively, it may prevent prescribed or cultural fire operations due to a mischaracterization of actual fire hazard or risk (Hollingsworth et al. 2012). Likewise, underpredicting potential fire behavior can lead to delayed advisories and the misallocation of suppression resources, putting firefighters, communities, and resource values at risk (Jolly 2007; Jenkins et al. 2012). A response to this uncertainty has been the development of unofficial FBP System fuel types (e.g., modified versions of C-3 and C-7 [C-7b] fuel types) that are used internally for wildfire operations but have not been formally documented (Perrakis and Eade 2015; BCWS, personal communications).

**Fig. 6:**
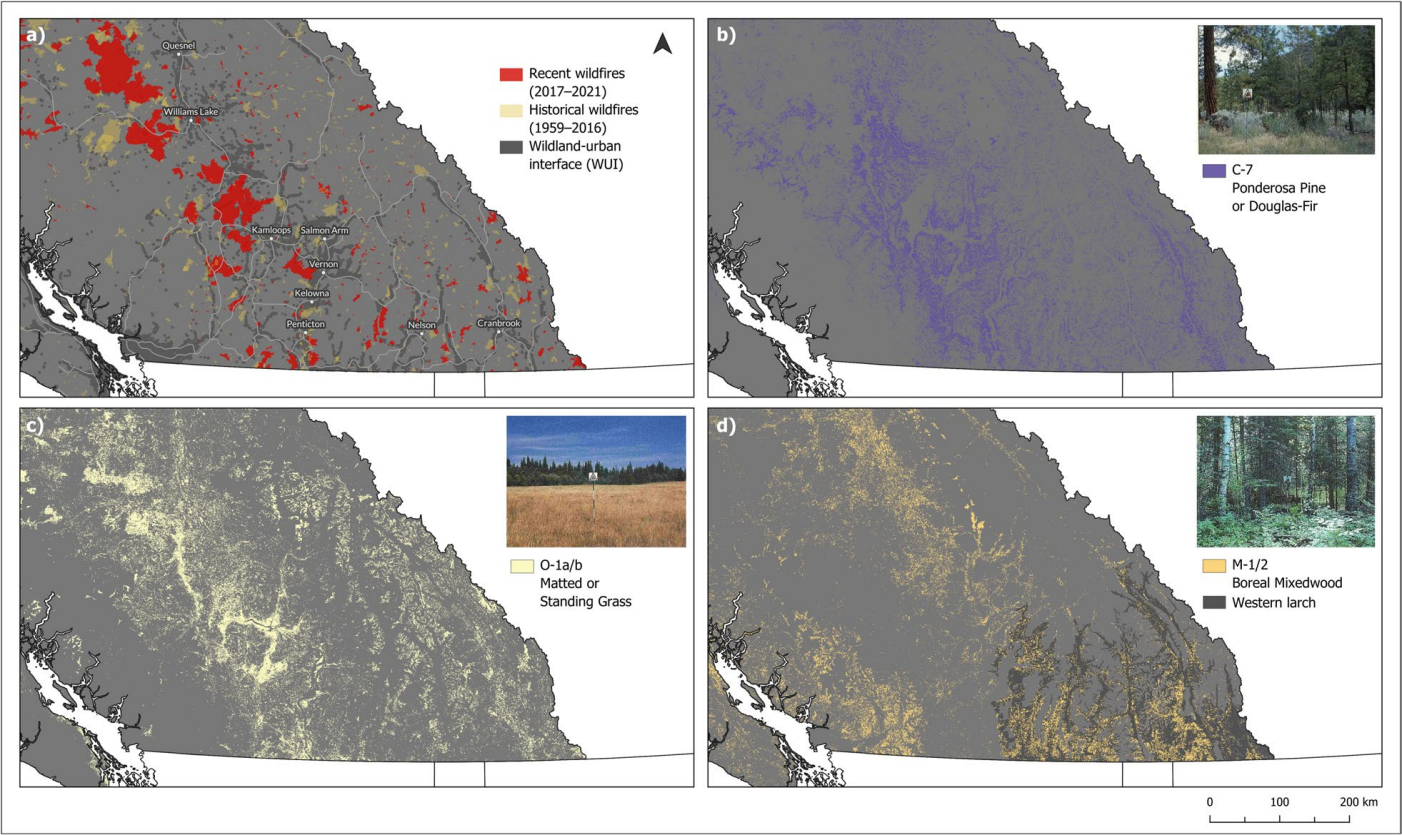
**a** Recent (2017–2021) and historical (1959–2016) wildfires (BC Wildfire Service 2023a), the wildland-urban interface (BC Wildfire Service 2023b), and major roads in interior British Columbia. Provincial fuel types with recurring mismatches: **b** C-7 ponderosa pine or Douglas-fir, **c** O-1 matted or standing grass, and **d** M-1/2 boreal mixedwood, with the extent of Western larch (Hamann et al. 2005). Reference Image Source: “FBP Fuel Type Description Webpage (https://cwfis.cfs.nrcan.gc.ca/background/fueltypes/c1).” Canadian Forest Service, Natural  Resources Canada. Reproduced with the permission of the Department of Natural Resources, 2023

More broadly, provincial fuel type maps are often used to model and prioritize fuel reduction treatments. In BC, the majority of these treatments occur in close proximity to communities, in the expanding wildland-urban interface (WUI) (Radeloff et al. 2018; Erni et al. 2021) (Fig. 6). Near dry interior forests, the WUI is characterized by dense fuel accumulations that are poorly represented by the FBP System fuel types. In this study, dense fuel accumulations in dry forest types were consistently classified as more open conditions (e.g., C-7) in provincial and national fuel type maps, based primarily on leading species composition. When remotely sensed fuel type maps are used to model fire behavior and plan mitigative fuel treatments, fuel typing mismatches may result in a failure to identify and target at-risk fuel conditions. Instead, such exercises may result in the placement of fuel treatments in sub-optimal locations for wildfire risk reduction. As a result, exposed communities will be challenged to identify and mitigate fuels and to accurately represent the potential benefits of treating fuels in the broader context of wildfire risk, community watershed protection, and smoke and carbon emissions.

Finally, in a research context, FBP System fuel types are used to understand, model, and predict fire behavior across vegetation types and structural conditions, often in the absence of plot-level fire behavior data (Parisien et al. 2019; Coogan et al. 2021). The limited availability of relevant fuel attribute data and FBP System fuel types often requires a “make it fit” approach, whereby expert advice is used to classify fuels. For example, to derive fuel type maps for burn probability modeling in the Columbia Mountains, Parisien et al. (2013) relied on expert advice to classify mature cedar-hemlock fuels as C-5 (Red and White Pine), a fuel type from eastern Canada. This classification, which is also commonly used in wildfire operations (BCWS, personal communications), was applied despite significant differences in fuel structure and fire intensity, under the assumption that these two vegetation complexes would produce similar rates of fire spread. Low confidence in fuel typing limits the ability of existing stand- and landscape fire-vegetation models to accurately represent fire behavior in terms of fire rate of spread, expected flame length and fireline intensity, and the potential for crown fire initiation and spread. At a landscape scale, the propagation of fire to neighboring patches is dependent on the composition and configuration of fuels. Errors in stand-level fuel typing thus propagate to introduce cascading uncertainties in predictions of wildfire spread and intensification across landscapes, limiting broad-scale research.

### Improving fuel characterizations

In this study, we have highlighted the need for improved forest inventory field data and fuel typing systems that represent the range of fuelbeds encountered across scales in the fire-prone forests of interior BC. The need for an enhanced fuel inventory was identified during the original classification of FBP System fuel types in BC. Hawkes et al. (1995) suggested that future fuel characterizations would require more comprehensive sampling of spatially explicit fuel data and a much-improved translation of inventory data to FBP System fuel types. We emphasize the critical importance of also understanding management and disturbance history, including recent insect outbreaks and severe wildfire seasons, which alter current and future fuels and challenge fire behavior prediction. Importantly, these challenges for fuel characterization are not unique to BC, but are common across geographies and fuel modeling systems (Keane 2013). Therefore, our recommendations for improving fuel characterizations are broadly relevant to inform the science and management of fire-prone ecosystems.

Inventories of forest and fuel attributes at broad spatial scales are challenged by canopy concealment, fuelbed complexity beneath the canopy, and fuel type diversity and variability (Keane et al. 2001). Of the attributes frequently used to characterize fuels and predict fire behavior, canopy stand height, canopy cover, and canopy species and proportion currently exist in provincial forest inventory and can be readily improved through satellite remote sensing and ground truthing (Matasci et al. 2018; Shang et al. 2020; Hermosilla et al. 2022). Overstory structural attributes, including canopy base height and canopy bulk density, are not currently represented in provincial forest inventories, but can be approximated using Light Detection and Ranging (LiDAR), if calibrated to sufficiently dense field plot data (Riaño et al. 2004; Andersen et al. 2005; Zhao et al. 2011; Jeronimo et al. 2018; Engelstad et al. 2019; Chamberlain et al. 2021, 2023). The great heterogeneity of subcanopy attributes describing surface and ladder fuels cannot currently be derived from remote sensing products and will necessitate applications of existing field inventory data (e.g., Hanes et al. 2021) and the development of new field-based fuel inventories (e.g., Phelps et al. 2022) to train products. Thus, fuel attribute data can be enhanced by using remote sensing products and reduced reliance on manual photointerpretation, but will require a significant complementary focus on field surveys and plot-level data.

Fundamentally, improved fuelbed characterizations will require a different fuel typing approach to represent the diversity of fuels associated with disturbed and managed forests. A *fuel classification* approach would better represent the full range of surface, ladder, and canopy fuel conditions observed in the field and would lead to much-improved simulation of expected fire behavior. The ability to de-couple and inventory surface, ladder, and canopy fuels at stand, regional, provincial, and national scales would better facilitate characterizations of complex and ephemeral fuel structures. For example, the Fuel Characteristic Classification (FCCS) system in the USA classifies fuels in six horizontal strata to catalog and represent extant fuel conditions. These fuel conditions can then serve as model inputs for fire behavior, fire effects, and fuel treatment efficacy modeling (Prichard et al. 2013, 2019). The photo-load sampling technique developed by Keane and Dickinson (2007) similarly provides a means of rapidly assaying surface fuels using graduated visual templates in the field. A similar application of techniques like these across BC would require a substantial effort in data collection and fuelbed typing to sufficiently represent the fuels associated with interior forest types.

Ongoing developments to the CFFDRS include a transition toward an attribute-based fuel typing approach (Canadian Forest Service Fire Danger Group 2021) and may begin to address the fundamental limitation of the current *fuel association* approach. In addition to this transition, improving fuel characterizations also necessitates a focus on fuels and fire behavior data to parameterize next-generation models. Specifically, improved fuel characterizations would benefit from direct measurements of fuels and fire behavior, across a range of fire weather, ignition, and topographic conditions, in the ecosystems for which fuels and fire behavior are being modeled. Although the CFFDRS was developed based on experimental crown fires, future data collection will likely need to rely on wild and prescribed fires due to the magnitude of data required to characterize modern-day shifting dynamics. To do so would require a coordinated effort in the systematic collection, storage, and integration of observational fuels and fire behavior data using field and remotely sensed approaches (e.g., Perrakis et al. 2014; Hart et al. 2021; Phelps et al. 2022). Simply put, the relationship between fuels and fire behavior cannot be understood or predicted without data for the systems we seek to represent.

## Conclusions

Our findings identified consistently low agreement in fuel typing for provincial and national data, relative to field-based classification. We identified frequent mismatches for (i) dry interior ecosystems, (ii) mixedwood and deciduous fuel types, and (iii) post-harvesting conditions. In many cases, fuelbeds without an applicable fuel type existed in the past but were not represented by the FBP System at the time of development (e.g., dry, mixedwood, and decidious forests). In other cases, fuelbeds present novel combinations of fuel conditions that did not exist in the past, or occurred at more localized scales than are observed today (e.g., modern post-harvesting conditions, severe insect outbreaks, re-burns). These mismatches constrain fundamental fire research and management decisions, including informing wildfire operations and fuel management.

We further identified the low accuracy and availability of forest inventory data and the limited applicability of the FBP System to interior BC as factors underpinning fuel typing mismatches. In the pursuit of a more universal and generalizable fuel description system, Keane (2013) argued that we are well advised to look to the future and not to the past. He suggested that expanded effort in basic research on wildland fuel science and fire behavior modeling is required to design fuel description systems that can meet contemporary needs. In BC, this will require a new approach that includes direct measurements of critical fuel attributes, broad fuel classifications that represent extant fuel conditions, and improved models to represent the variation in twenty-first-century fire behavior.

### Supplementary Information

**Additional file 1:** **Appendix A. Appendix B: Figure S1, Table S1, Table S2.**

## Data Availability

The provincial fuel typing data is available from the BC Data Catalogue [https://catalogue.data.gov.bc.ca/dataset/bc-wildfire-fire-fuel-types-public]. The national fuel typing data is available from the Canadian Forest Service [https://cwfis.cfs.nrcan.gc.ca/downloads/fuels/development/Canadian_Forest_FBP_Fuel_Types/Canadian_Forest_FBP_Fuel_Types_Metadata_v20191114.pdf]. The 2018 provincial decision tree script is available on GitHub [https://github.com/gagreene/BC_CFFBPS_FuelTyping_Tool]. The data and scripts generated during the study are available from the corresponding author on reasonable request.

## Abbreviations

BC: British Columbia
BCWS: British Columbia Wildfire Service
CFFDRS: Canadian Forest Fire Danger Rating System
CFS: Canadian Forest Service
FBP: Fire Behavior Prediction
GIS: Geographic Information System
LiDAR: Light Detection and Ranging
VRI: Vegetation Resource Inventory
