# Supplementary material for: Fuel types misrepresent forest structure and composition in interior British Columbia: a way forward
Source: Fire Ecol. 2024 Feb 7;20(1):15. doi: 10.1186/s42408-024-00249-z (PMC10847212; doi:10.1186/s42408-024-00249-z)
Supplement: Supplementary file 1 — Additional file 1: Appendix A. Appendix B: Figure S1, Table S1, Table S2. [file 42408_2024_249_MOESM1_ESM.pdf]

Supplementary Material

Appendix A.

Field Methods: Survey123 Data Collection Form

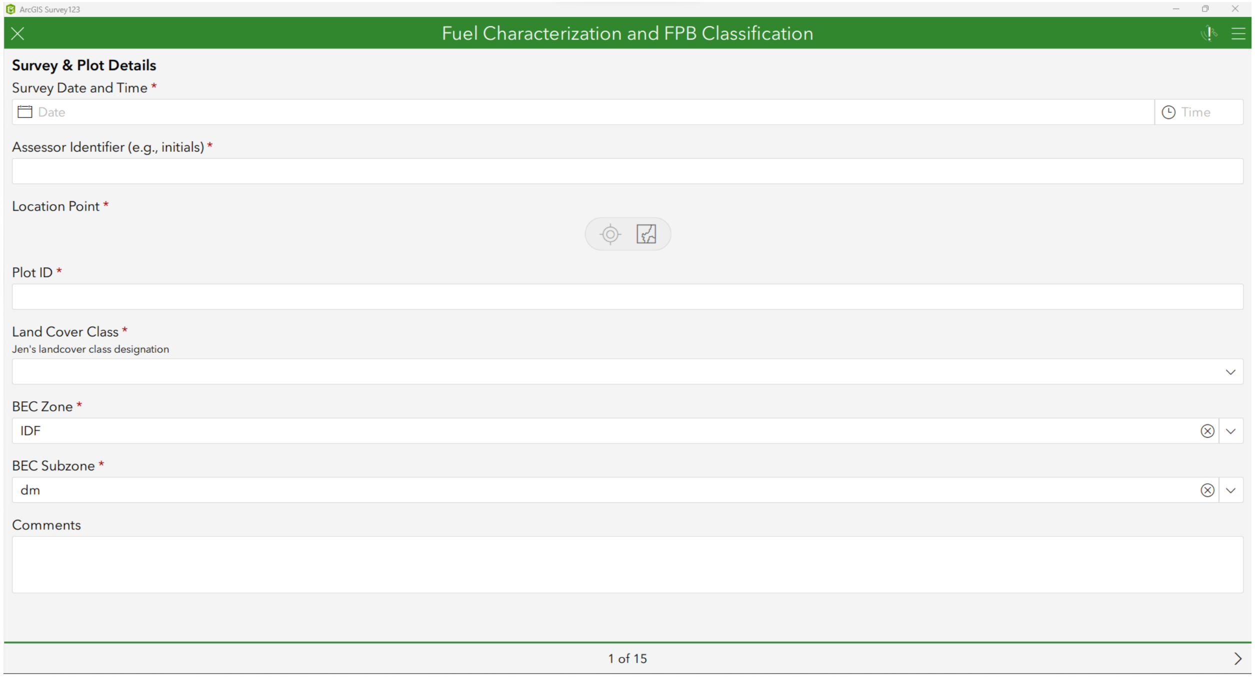

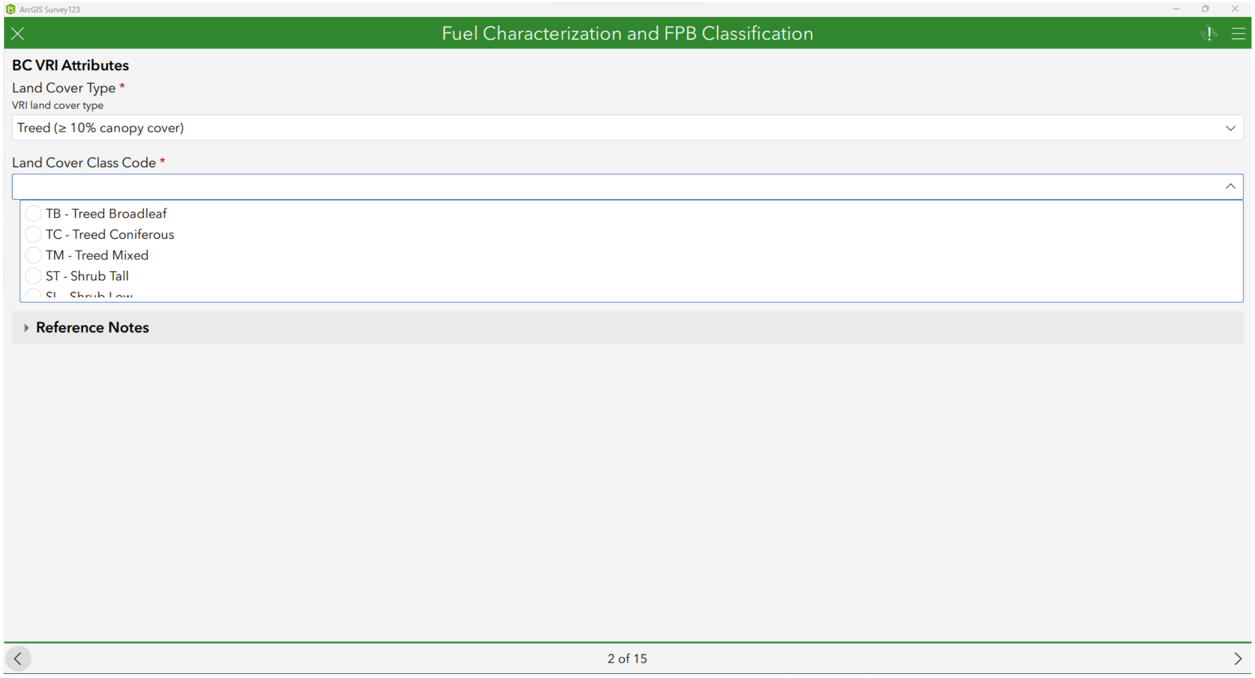

Supplementary Material

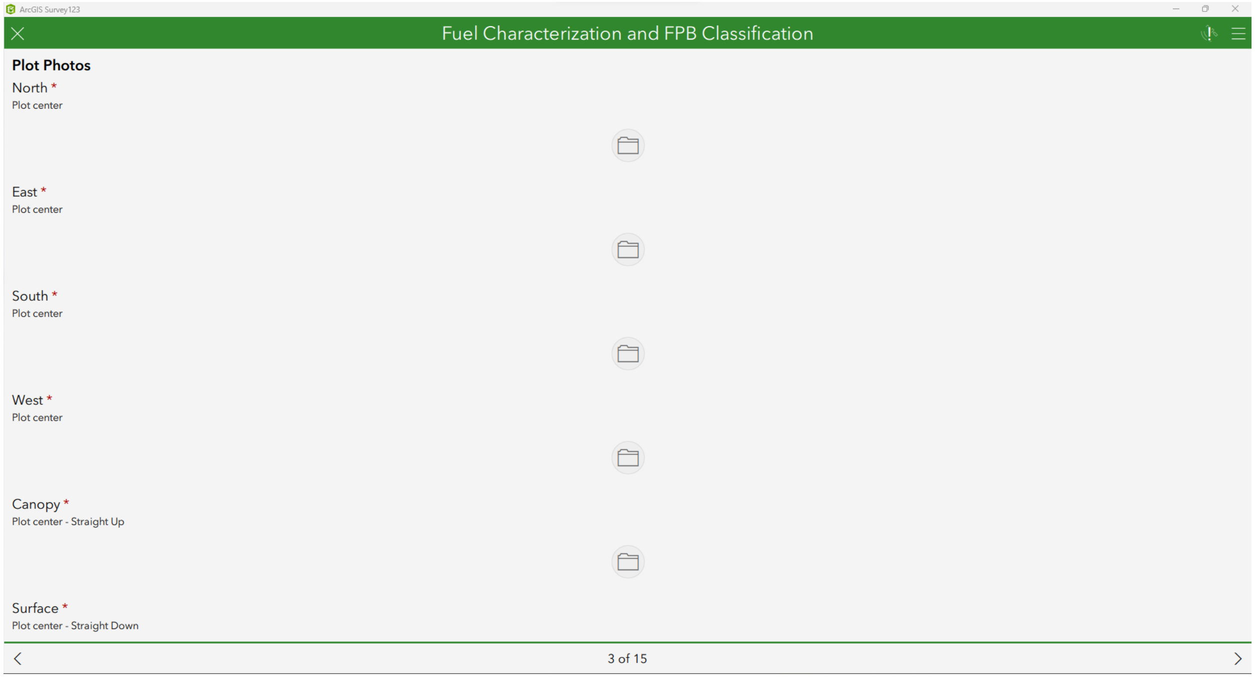
ArcGIS Survey123

Fuel Characterization and FPB Classification

Plot Photos

North *
Plot center

East *
Plot center

South *
Plot center

West *
Plot center

Canopy *
Plot center - Straight Up

Surface *
Plot center - Straight Down

3 of 15

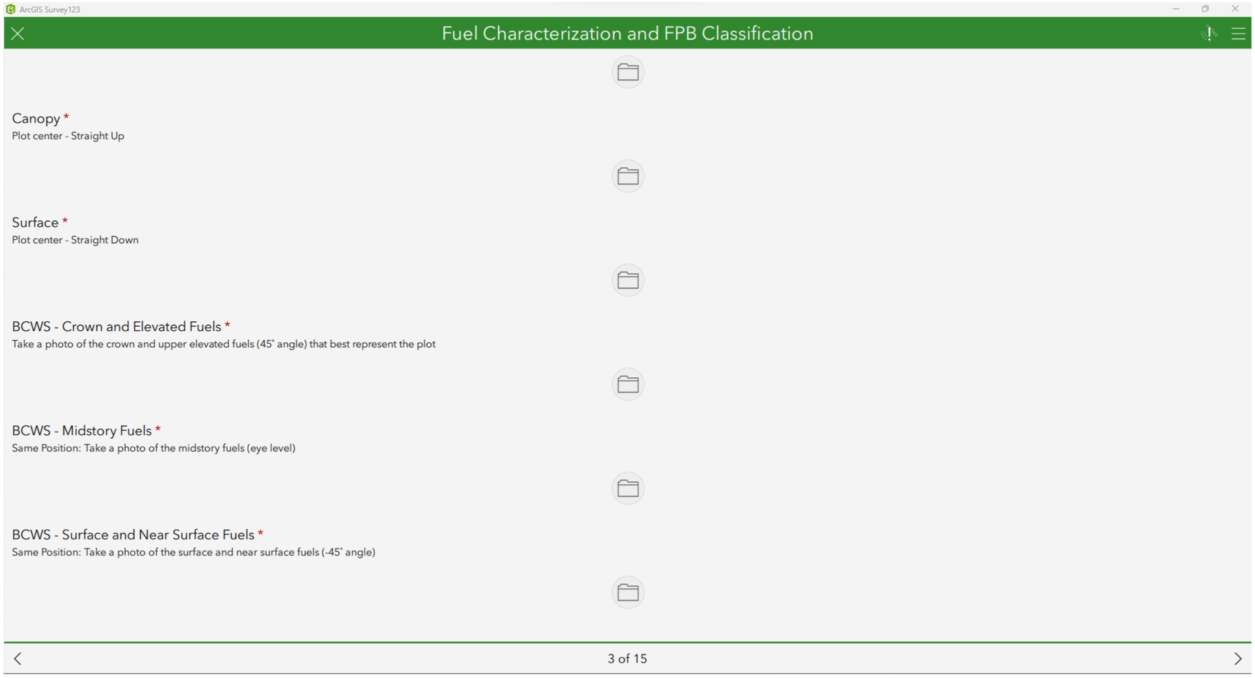
ArcGIS Survey123

Fuel Characterization and FPB Classification

Canopy *
Plot center - Straight Up

Surface *
Plot center - Straight Down

BCWS - Crown and Elevated Fuels *
Take a photo of the crown and upper elevated fuels (45° angle) that best represent the plot

BCWS - Midstory Fuels *
Same Position: Take a photo of the midstory fuels (eye level)

BCWS - Surface and Near Surface Fuels *
Same Position: Take a photo of the surface and near surface fuels (-45° angle)

3 of 15

Supplementary Material

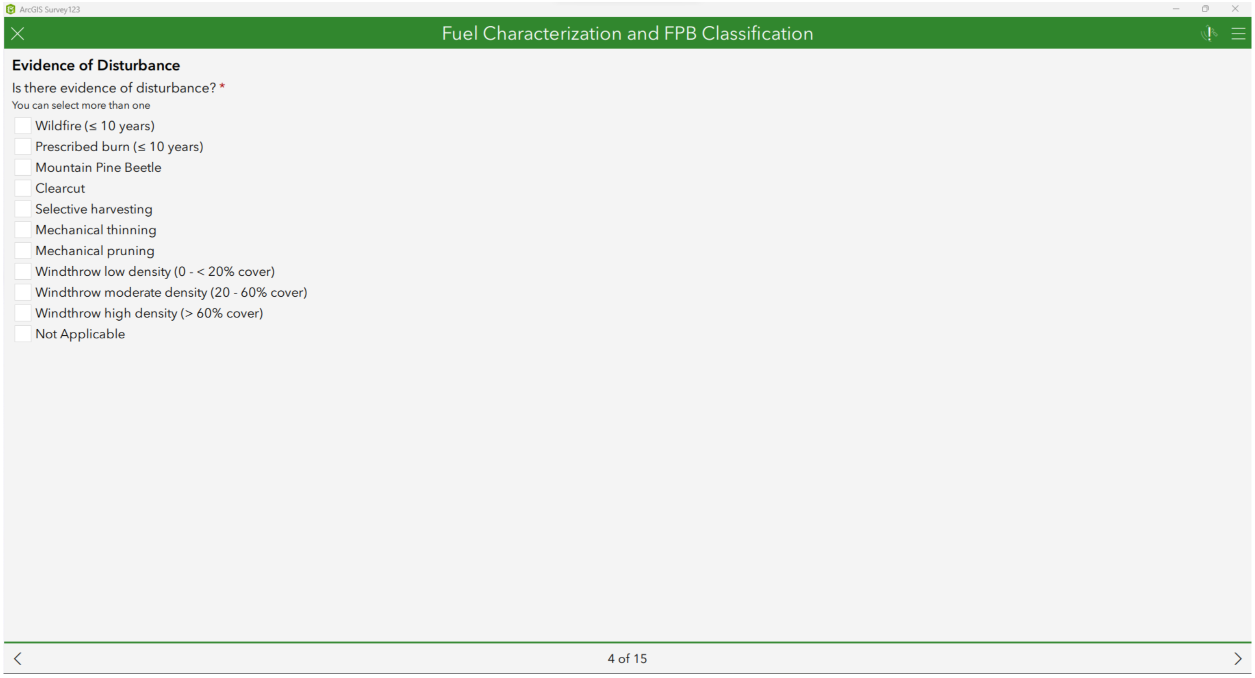
ArcGIS Survey123

Fuel Characterization and FPB Classification

Evidence of Disturbance

Is there evidence of disturbance? *

You can select more than one

- Wildfire (≤ 10 years)
- Prescribed burn (≤ 10 years)
- Mountain Pine Beetle
- Clearcut
- Selective harvesting
- Mechanical thinning
- Mechanical pruning
- Windthrow low density (0 - < 20% cover)
- Windthrow moderate density (20 - 60% cover)
- Windthrow high density (> 60% cover)
- Not Applicable

4 of 15

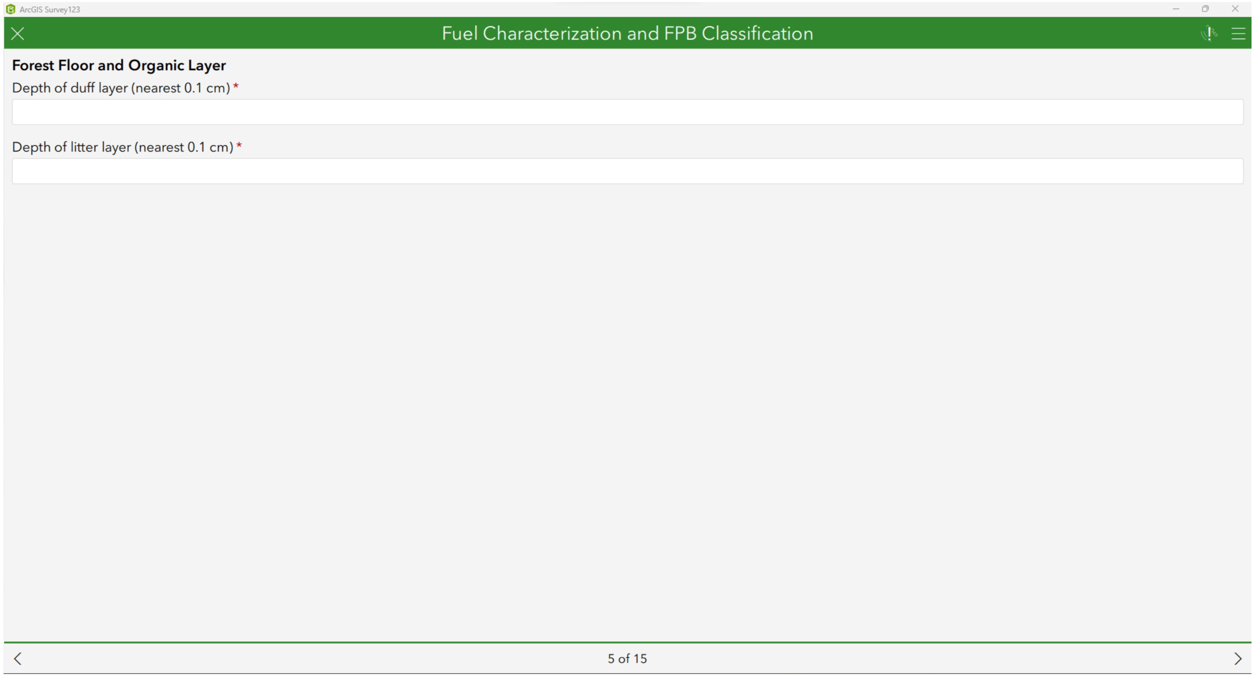
ArcGIS Survey123

Fuel Characterization and FPB Classification

Forest Floor and Organic Layer

Depth of duff layer (nearest 0.1 cm) *

Depth of litter layer (nearest 0.1 cm) *

5 of 15

Supplementary Material

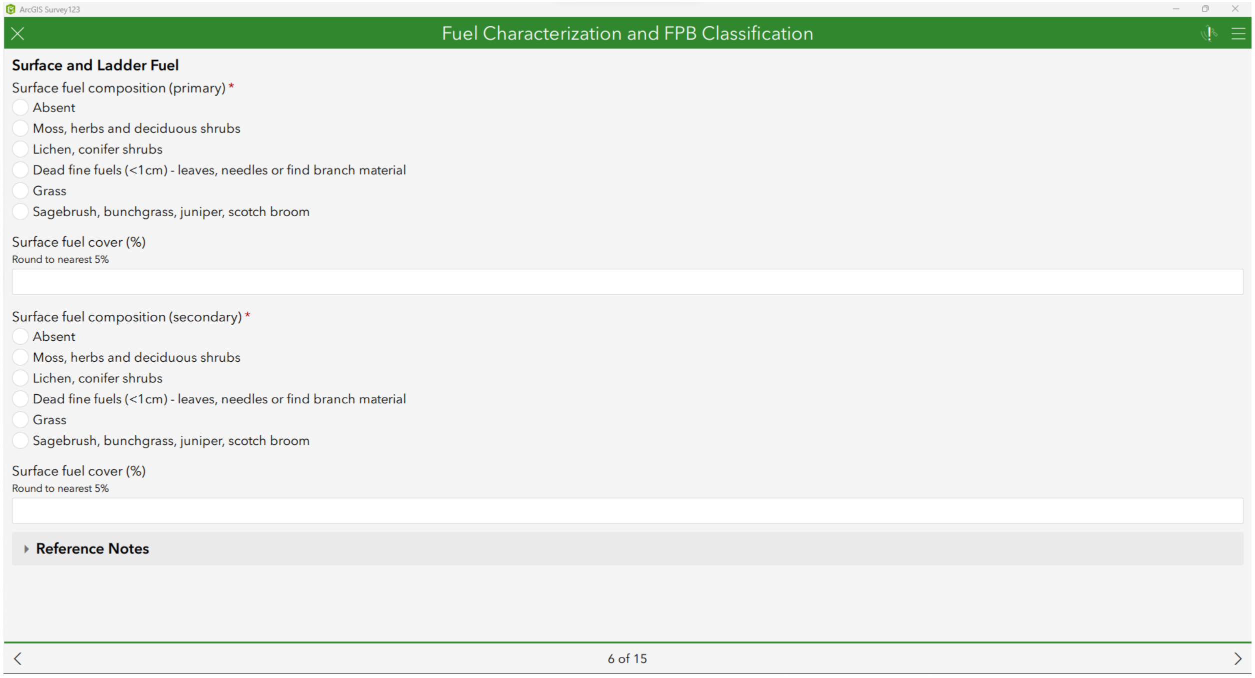

ArcGIS Survey123

Fuel Characterization and FPB Classification

Surface and Ladder Fuel

Surface fuel composition (primary) *

- Absent
- Moss, herbs and deciduous shrubs
- Lichen, conifer shrubs
- Dead fine fuels (<1cm) - leaves, needles or find branch material
- Grass
- Sagebrush, bunchgrass, juniper, scotch broom

Surface fuel cover (%)

Round to nearest 5%

Surface fuel composition (secondary) *

- Absent
- Moss, herbs and deciduous shrubs
- Lichen, conifer shrubs
- Dead fine fuels (<1cm) - leaves, needles or find branch material
- Grass
- Sagebrush, bunchgrass, juniper, scotch broom

Surface fuel cover (%)

Round to nearest 5%

Reference Notes

6 of 15

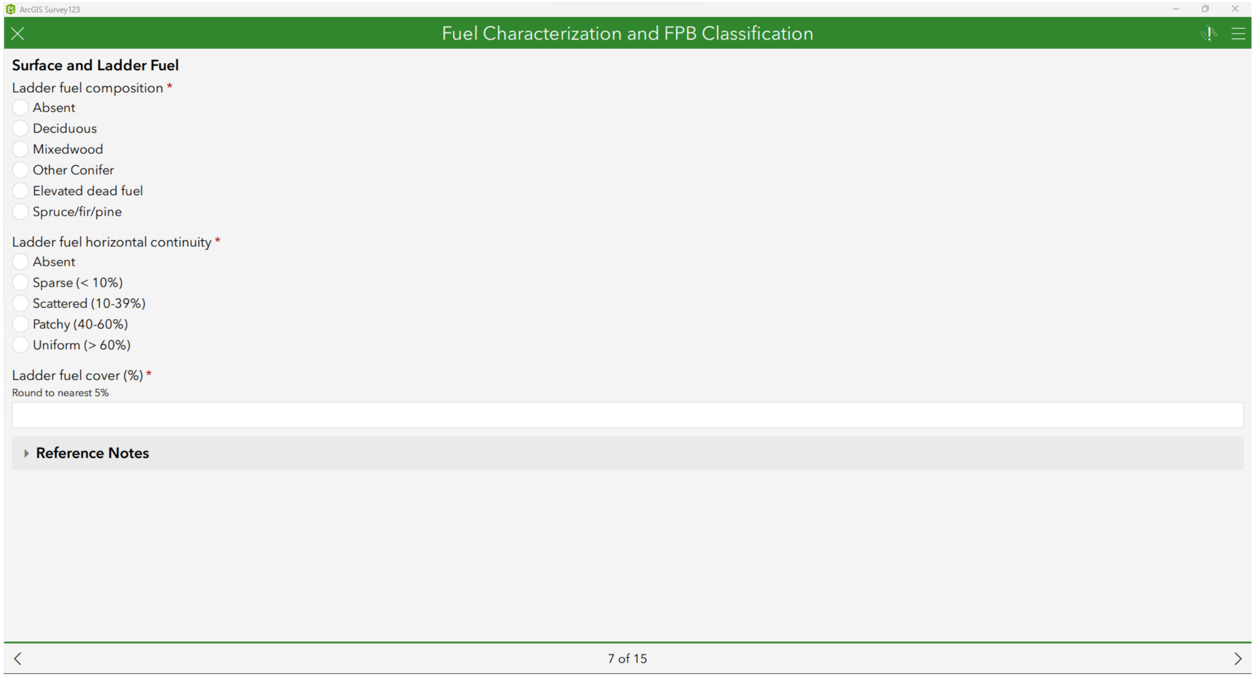

ArcGIS Survey123

Fuel Characterization and FPB Classification

Surface and Ladder Fuel

Ladder fuel composition *

- Absent
- Deciduous
- Mixedwood
- Other Conifer
- Elevated dead fuel
- Spruce/fir/pine

Ladder fuel horizontal continuity *

- Absent
- Sparse (< 10%)
- Scattered (10-39%)
- Patchy (40-60%)
- Uniform (> 60%)

Ladder fuel cover (%) *

Round to nearest 5%

Reference Notes

7 of 15

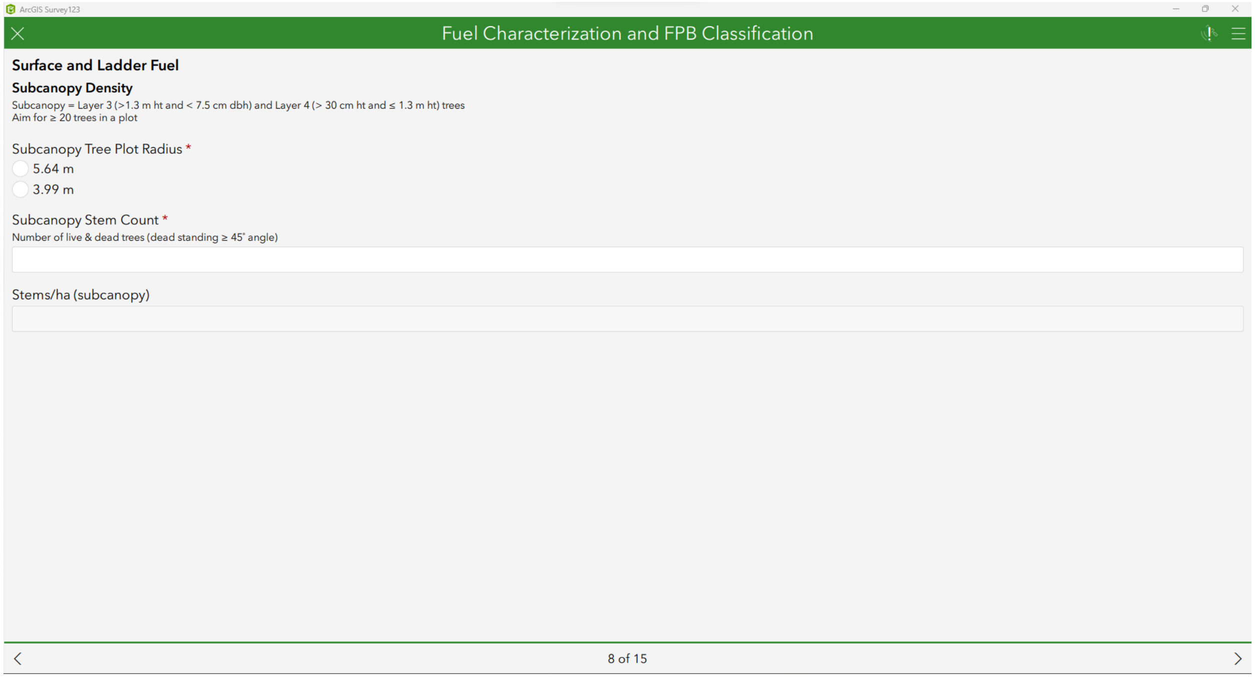

ArcGIS Survey123

Fuel Characterization and FPB Classification

Surface and Ladder Fuel

Subcanopy Density

Subcanopy = Layer 3 (>1.3 m ht and < 7.5 cm dbh) and Layer 4 (> 30 cm ht and ≤ 1.3 m ht) trees
Aim for ≥ 20 trees in a plot

Subcanopy Tree Plot Radius *

- 5.64 m
- 3.99 m

Subcanopy Stem Count *

Number of live & dead trees (dead standing ≥ 45° angle)

Stems/ha (subcanopy)

8 of 15

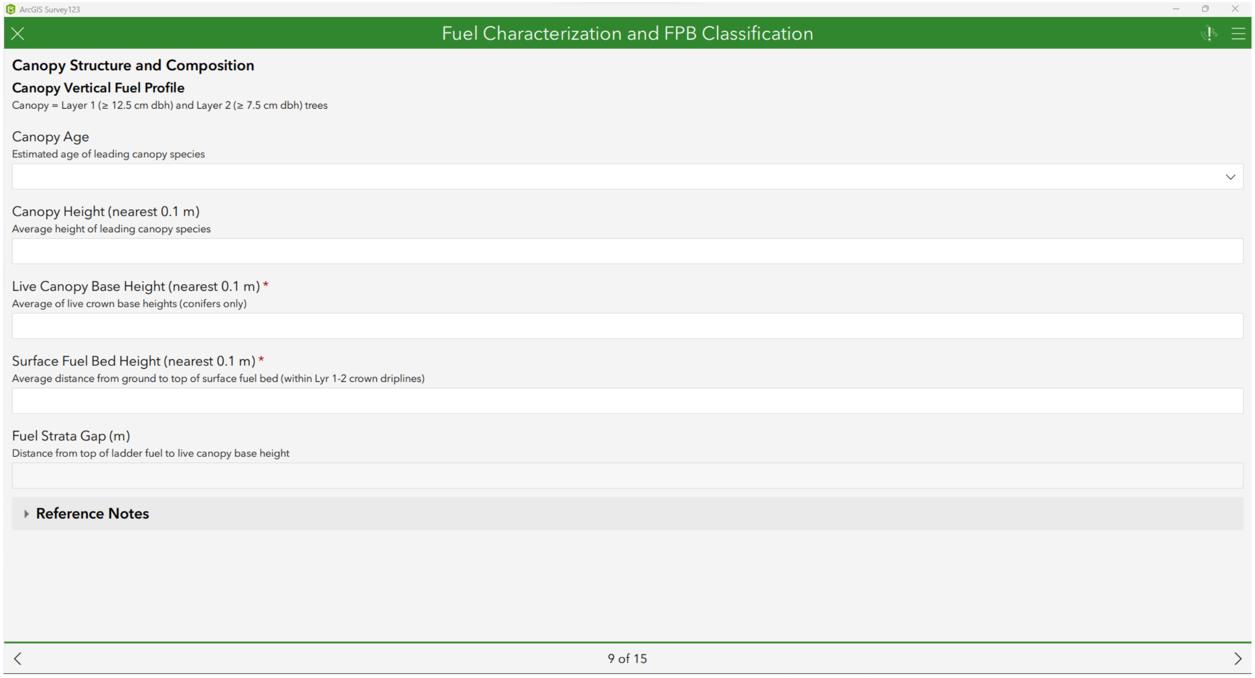

ArcGIS Survey123

Fuel Characterization and FPB Classification

Canopy Structure and Composition

Canopy Vertical Fuel Profile

Canopy = Layer 1 (≥ 12.5 cm dbh) and Layer 2 (≥ 7.5 cm dbh) trees

Canopy Age

Estimated age of leading canopy species

Canopy Height (nearest 0.1 m)

Average height of leading canopy species

Live Canopy Base Height (nearest 0.1 m) *

Average of live crown base heights (conifers only)

Surface Fuel Bed Height (nearest 0.1 m) *

Average distance from ground to top of surface fuel bed (within Lyr 1-2 crown driplines)

Fuel Strata Gap (m)

Distance from top of ladder fuel to live canopy base height

Reference Notes

9 of 15

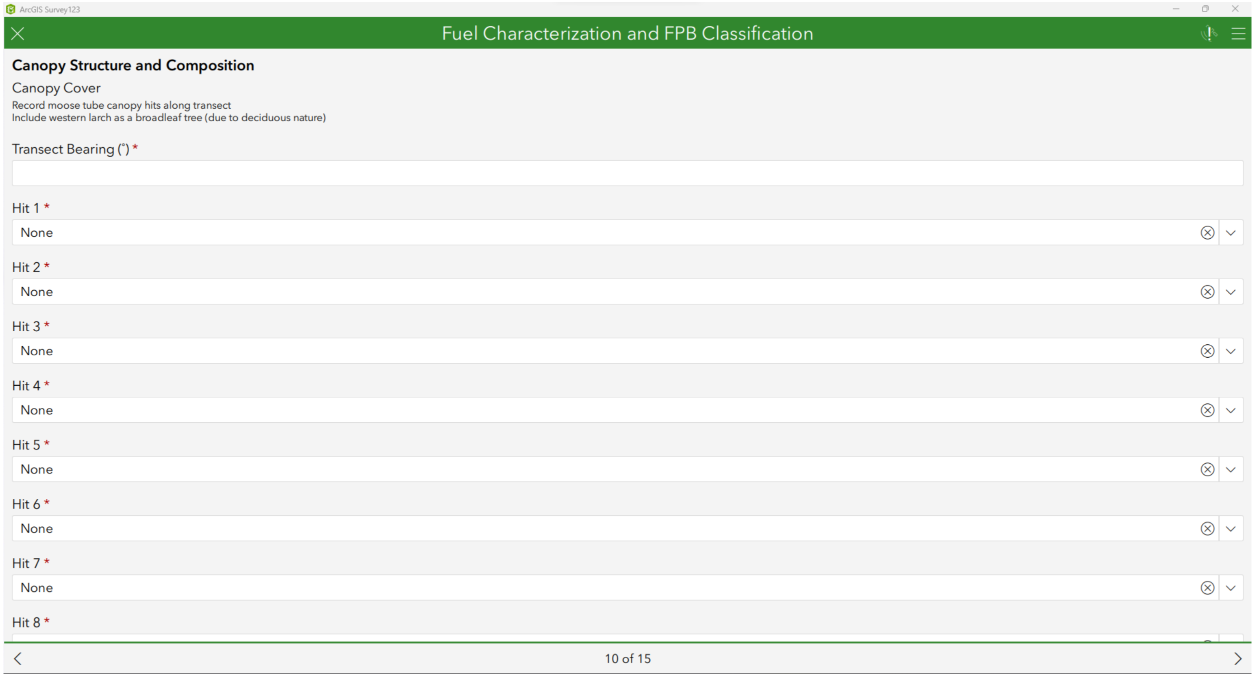

ArcGIS Survey123

Fuel Characterization and FPB Classification

Canopy Structure and Composition

Canopy Cover

Record moose tube canopy hits along transect
Include western larch as a broadleaf tree (due to deciduous nature)

Transect Bearing (°) *

Hit 1 *
None

Hit 2 *
None

Hit 3 *
None

Hit 4 *
None

Hit 5 *
None

Hit 6 *
None

Hit 7 *
None

Hit 8 *

10 of 15

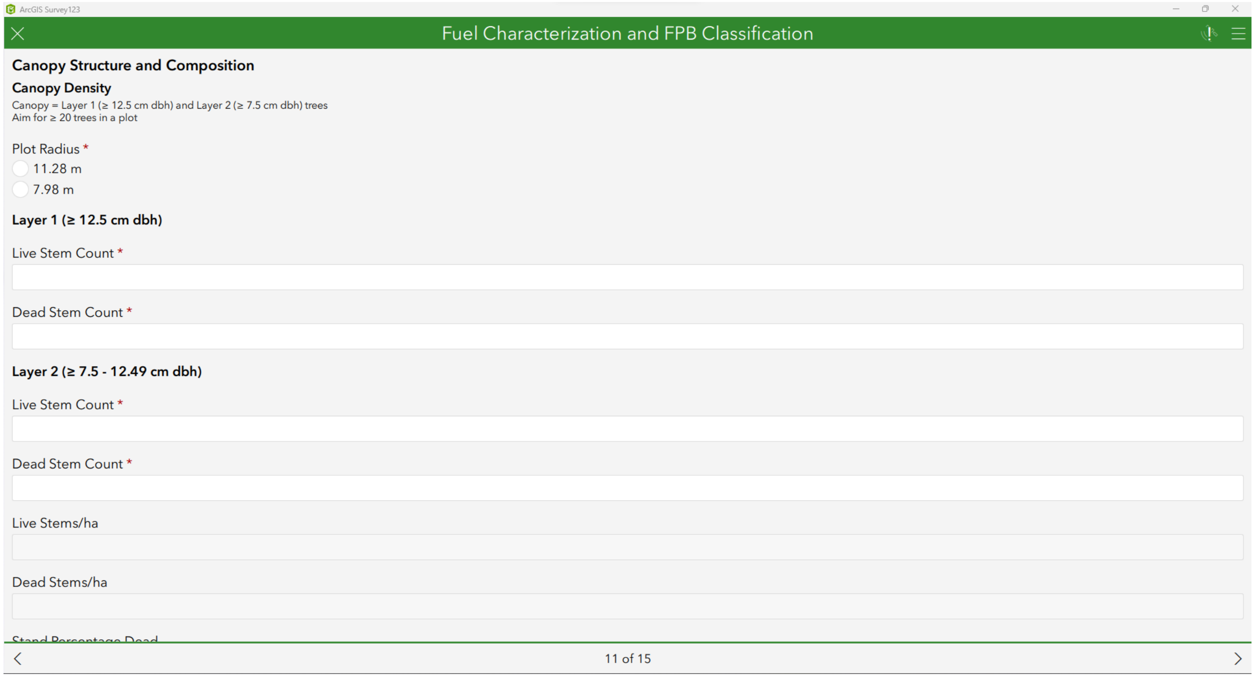

ArcGIS Survey123

Fuel Characterization and FPB Classification

Canopy Structure and Composition

Canopy Density

Canopy = Layer 1 (≥ 12.5 cm dbh) and Layer 2 (≥ 7.5 cm dbh) trees
Aim for ≥ 20 trees in a plot

Plot Radius *
11.28 m
7.98 m

Layer 1 (≥ 12.5 cm dbh)

Live Stem Count *

Dead Stem Count *

Layer 2 (≥ 7.5 - 12.49 cm dbh)

Live Stem Count *

Dead Stem Count *

Live Stems/ha

Dead Stems/ha

Stand Percentage Dead

11 of 15

ArcGIS Survey123

Fuel Characterization and FPB Classification

Canopy Structure and Composition

Canopy Composition & Basal Area

Live trees only
List species in order of dominance (based on number of "in" trees)

Prism Basal Area Factor *

Choose BAF to ensure there are 8-10 "in" trees

- 1
- ● 2
- 3
- 4

Species 1 *

Not Applicable

Count ("in" trees)*

0

Species 2 *

Not Applicable

Count ("in" trees)*

0

Species 3 *

Not Applicable

Count ("in" trees)*

0

Species 4 *

12 of 15

ArcGIS Survey123

Fuel Characterization and FPB Classification

Stand Health

Evidence of Vegetation Stress *

- ● None
- Insect
- Disease
- Climate Stress
- Competition

13 of 15

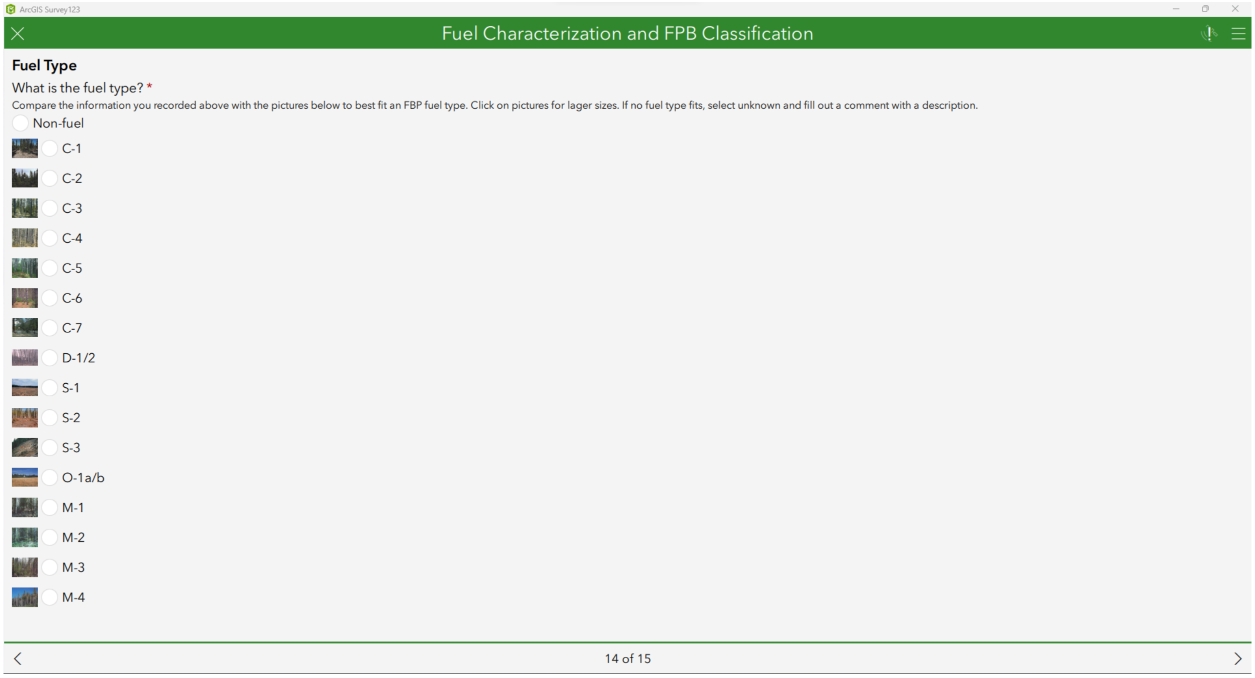
ArcGIS Survey123
Fuel Characterization and FPB Classification
Fuel Type
What is the fuel type? *
Compare the information you recorded above with the pictures below to best fit an FBP fuel type. Click on pictures for lager sizes. If no fuel type fits, select unknown and fill out a comment with a description.
Non-fuel
C-1
C-2
C-3
C-4
C-5
C-6
C-7
D-1/2
S-1
S-2
S-3
O-1a/b
M-1
M-2
M-3
M-4
14 of 15

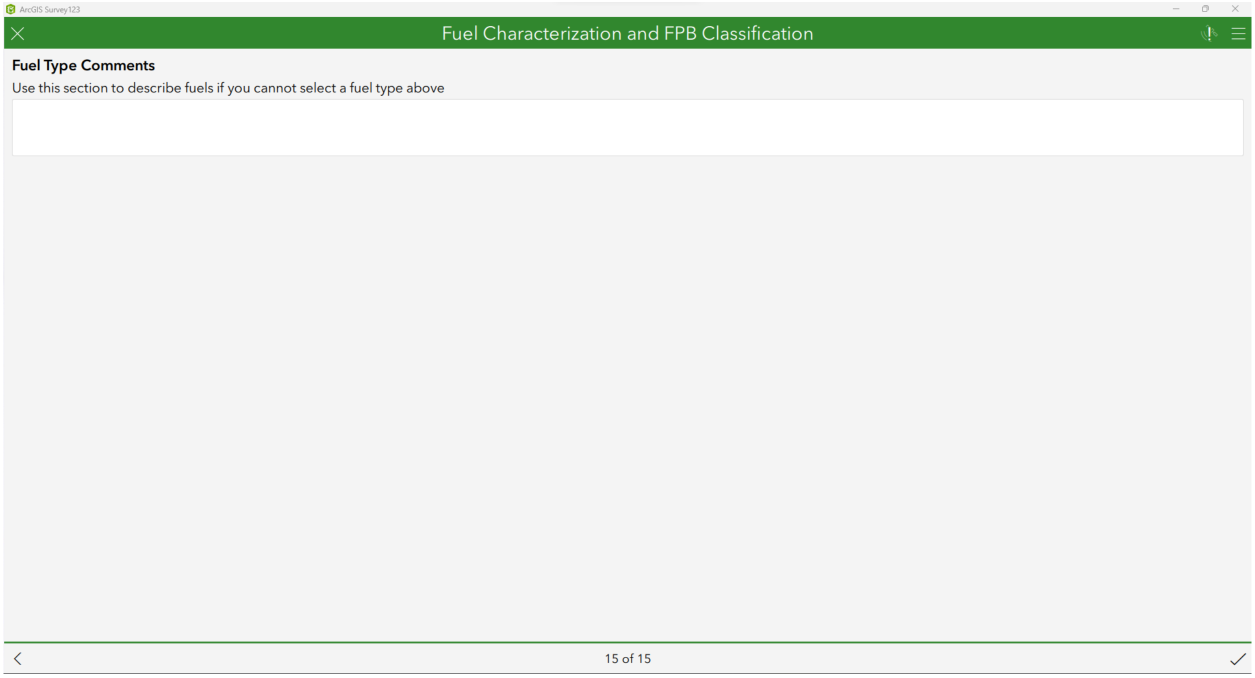
ArcGIS Survey123
Fuel Characterization and FPB Classification
Fuel Type Comments
Use this section to describe fuels if you cannot select a fuel type above
15 of 15

Appendix B.

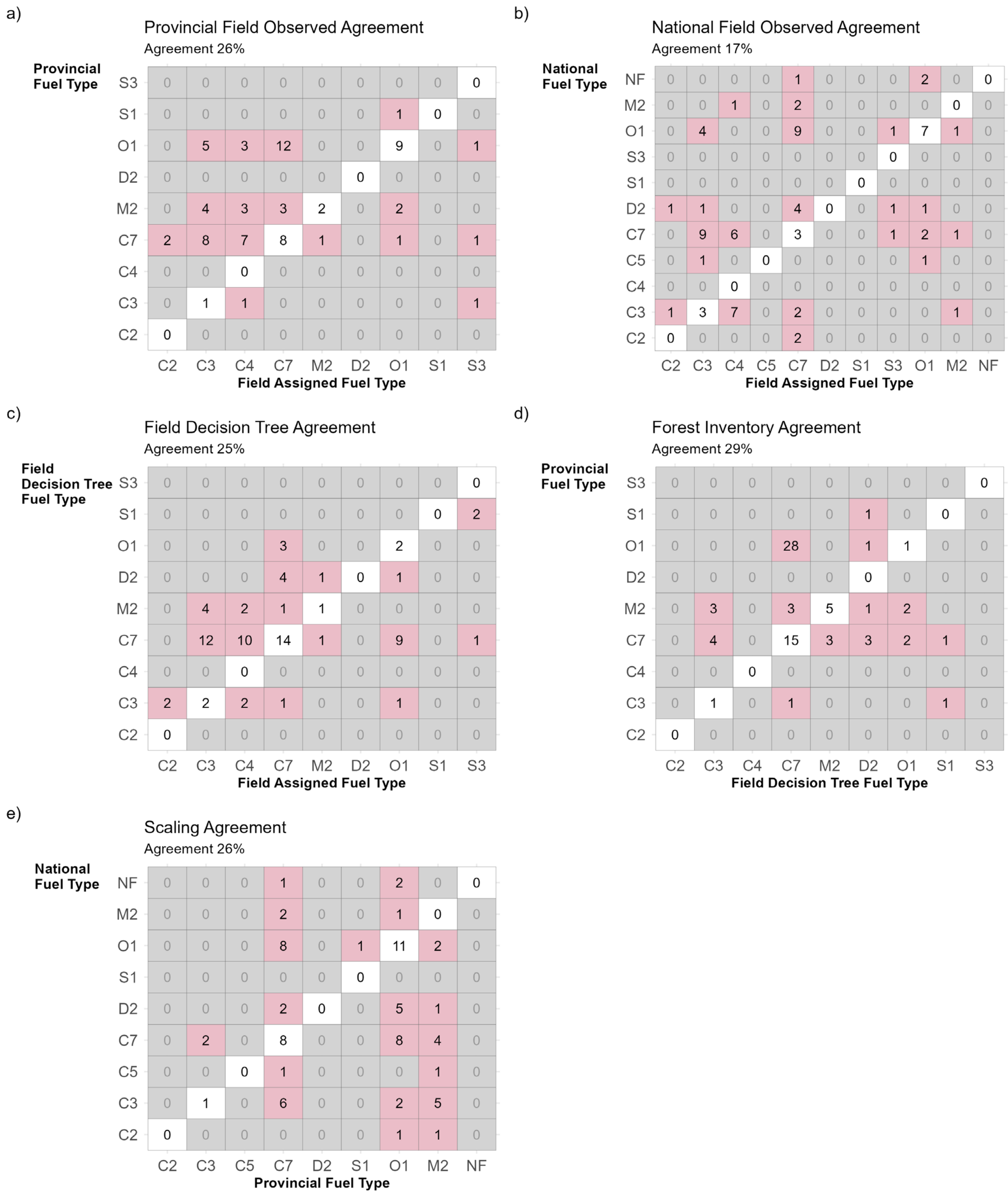

Figure S1. Confusion matrices showing mismatches between fuel typing data: **a)** provincial field observed agreement (provincial-field assigned), **b)** national field observed agreement (national-field assigned), **c)** field decision tree agreement (field decision tree-field assigned), **d)** forest inventory agreement (provincial-field decision tree), and **e)** scaling agreement (national-provincial).

Table S1. Fuel typing mismatches, decision tree process numbers, and decision criteria for field decision tree agreement (field assigned-field decision tree).

Mismatch	# Plots	Process #	Decision Criteria
C4-C7	8	27	Vegetated / Forested / Pure species stand / FD leading / Not logged or logging > 6 years / Species 1 HT >= 4m / Crown closure > 55% / Species 1 HT > 12m / Dry BEC zone or ICH (dry)
O1-C7	8	622	Vegetated / Non-forested / Not recently burned (> 10 years) / Logged (> 24 years) / BEC = dry IDF
C3-C7	5	31	Vegetated / Forested / Pure species stand / FD leading / Not logged or logging > 6 years / Species 1 HT >= 4m / Crown closure 26-55% / Interior dry BEC zone
C3-C7	4	223	Vegetated / Forested / Mixed species stand / Pure conifer (80-100%) / FD leading / Not logged or logging > 7 years / Species 1 HT >= 4 m / Crown closure 26-55% / Interior dry BEC zone
C7-D2	3	1618	Vegetated / Forested / Recently burned (< 11 years) / Conifer forest / Crown closure <= 40% / Burned within 2-6 years
C7-O1	3	96	Vegetated / Forested / Pure species stand / FD leading / Not logged or logging > 6 years / Species 1 HT >= 4m / Crown closure < 26% / Not CWH, CDF, MH or ICH (wet) BEC zone
C4-C7	2	31	Vegetated / Forested / Pure species stand / FD leading / Not logged or logging > 6 years / Species 1 HT >= 4m / Crown closure 26-55% / Interior dry BEC zone
C3-M2	2	122	Vegetated / Forested / Mixed species stand / Not logged or logging > 6 years / 41-65% conifer / FD leading / Not CWH, CDF or ICH BEC zone
S3-S1	2	310	Vegetated / Non-forested / Not recently burned (> 10 years) / Logged (<= 7 years) / Species 1 not P*, S*, B*, CW, YC, H*, FD
O1-D2	1	1621	Vegetated / Forested / Recently burned (< 11 years) / Mixedwood or deciduous / Burned within 2-10 years
C4-C3	1	16	Vegetated / Forested / Pure species stand / PLI or PJ leading / Not logged or logging > 8 years / Dense or open / Species 1 HT 4-12 m / Fully stocked (live + dead stems < 8000)
C2-C3	1	1522	Vegetated / Forested / Pure species stand / PLI or PJ leading / Not logged or logging > 8 years / Species 1 HT > 12 m / Crown closure > 40 / non-MPB closed mature pine stand
C3-C7	1	1540	Vegetated / Forested / Pure species stand / PY leading / Dense or open / Not logged or logged > 11 years / > 17 m height
O1-C7	1	1546	Vegetated / Forested / Pure species stand / PY leading / Sparse / Stand percent dead < 40% / Not logged or logging > 11 years
C4-C3	1	29	Vegetated / Forested / Pure species stand / FD leading / Not logged or logging > 6 years / Species 1 HT >= 4m / Crown closure > 55% / Species 1 HT = 4-12m / Dry BEC zone or ICH (dry) / Percent dead <= 34% or no dead trees
C3-C7	1	27	Vegetated / Forested / Pure species stand / FD leading / Not logged or logging > 6 years / Species 1 HT >= 4m / Crown closure > 55% / Species 1 HT > 12m / Dry BEC zone or ICH (dry)
C7-D2	1	94	Vegetated / Forested / Pure species stand / Deciduous-broadleaf or larch leading

M2-D2	1	94	Vegetated / Forested / Pure species stand / Deciduous-broadleaf or larch leading
C3-M2	1	114	Vegetated / Forested / Mixed species stand / Not logged or logging > 6 years / 41-65% conifer / PL, PLI, PLC, PJ or P leading / Stand open
C7-M2	1	1533	Vegetated / Forested / Mixed species stand / Not logged or logging > 6 years / 41-65% conifer / PY leading
C4-M2	1	122	Vegetated / Forested / Mixed species stand / Not logged or logging > 6 years / 41-65% conifer / FD leading / Not CWH, CDF or ICH BEC zone
C3-M2	1	140	Vegetated / Forested / Mixed species stand / Not logged or logging > 6 years / 41-65% conifer / SX or S leading / Not BWBS or SWB BEC zone / Stand open or dense / Stand in interior
C4-M2	1	166	Vegetated / Forested / Mixed species stand / Not logged or logging > 6 years / 65-80% conifer / FD leading / Not CWH, CDF or ICH (wet) BEC zone / Stand dense
M2-C7	1	168	Vegetated / Forested / Mixed species stand / Not logged or logging > 6 years / 65-80% conifer / FD leading / Not CWH, CDF or ICH (wet) BEC zone / Stand not dense
C3-C7	1	202	Vegetated / Forested / Mixed species stand / Pure conifer (80-100%) / PY leading / Not logged or logging > 7 years / Species 1 HT >= 4 m / Stand open or sparse
C2-C3	1	250	Vegetated / Forested / Mixed species stand / Pure conifer (80-100%) / S leading / Not logged or logging > 7 years / Species 1 = SX, SW or S / BEC not SBWS / Interior BEC zone / Stand open or sparse / Percent dead <= 34 or no dead trees / Species 2 not PL, PLI or P
C7-C3	1	620	Vegetated / Non-forested / Not recently burned (> 10 years) / Logged (> 24 years) / MS BEC zone
O1-C3	1	620	Vegetated / Non-forested / Not recently burned (> 10 years) / Logged (> 24 years) / MS BEC zone
S3-C7	1	622	Vegetated / Non-forested / Not recently burned (> 10 years) / Logged (> 24 years) / dry IDF BEC zone

See Perrakis et al. 2018 for decision tree and complete description of attributes, including species codes.

Table S2. Fuel typing mismatches, decision tree process numbers, and decision criteria for forest inventory agreement (field decision tree-provincial).

Mismatch	# Plots	Process #	Decision Criteria
C7-O1	9	622	Vegetated / Non-forested / Not recently burned (> 10 years) / Logged (> 24 years) / BEC = dry IDF
C7-O1	7	202	Vegetated / Forested / Mixed species stand / Pure conifer (80-100%) / PY leading / Not logged or logging > 7 years / Species 1 HT >= 4 m / Stand open or sparse
C7-O1	5	1546	Vegetated / Forested / Pure species stand / PY leading / Sparse / Stand percent dead < 40% / Not logged or logging > 11 years
C7-O1	4	31	Vegetated / Forested / Pure species stand / FD leading / Not logged or logging > 6 years / Species 1 HT >= 4m / Crown closure 26-55% / Interior dry BEC zone
C3-C7	2	1522	Vegetated / Forested / Pure species stand / PLI or PJ leading / Not logged or logging > 8 years / Species 1 HT > 12 m / Crown closure > 40 / Non-MPB closed mature pine stand
C3-M2	2	620	Vegetated / Non-forested / Not recently burned (> 10 years) / Logged (> 24 years) / BEC = MS
C7-O1	2	27	Vegetated / Forested / Pure species stand / FD leading / Not logged or logging > 6 years / Species 1 HT >= 4m / Crown closure > 55% / Species 1 HT > 12m / Dry BEC zone or ICH (dry)
D2-C7	2	1618	Vegetated / Forested / Recently burned (< 11 years) / Conifer forest / Crown closure <= 40% / Burned within 2-6 years
C3-C7	1	29	Vegetated / Forested / Pure species stand / FD leading / Not logged or logging > 6 years / Species 1 HT >= 4m / Crown closure > 55% / Species 1 HT = 4-12m / Dry BEC zone or ICH (dry) / Percent dead <= 34% or no dead trees
C3-C7	1	250	Vegetated / Forested / Mixed species stand / Pure conifer (80-100%) / S leading / Not logged or logging > 7 years / Species 1 = SX, SW or S / BEC not SBWS / Interior BEC zone / Stand not dense / Percent dead <= 34 or no dead trees / Species 2 not PL, PLI or P
C3-M2	1	16	Vegetated / Forested / Pure species stand / PLI or PJ leading / Not logged or logging > 8 years / Dense or open / Species 1 HT 4-12 m / Fully stocked (live + dead stems < 8000)
C7-C3	1	27	Vegetated / Forested / Pure species stand / FD leading / Not logged or logging > 6 years / Species 1 HT >= 4m / Crown closure > 55% / Species 1 HT > 12m / Dry BEC zone or ICH (dry)
C7-M2	1	27	Vegetated / Forested / Pure species stand / FD leading / Not logged or logging > 6 years / Species 1 HT >= 4m / Crown closure > 55% / Species 1 HT > 12m / Dry BEC zone or ICH (dry)
C7-M2	1	31	Vegetated / Forested / Pure species stand / FD leading / Not logged or logging > 6 years / Species 1 HT >= 4m / Crown closure 26-55% / Interior dry BEC zone
C7-M2	1	168	Vegetated / Forested / Mixed species stand / Not logged or logging > 6 years / 65-80% conifer / FD leading / Not CWH, CDF or ICH (wet) BEC zone / Stand not dense

C7-O1	1	223	Vegetated / Forested / Mixed species stand / Pure conifer (80-100%) / FD leading / Not logged or logging > 6 years / Species 1 HT >= 4 m / Crown closure 26-55% / Interior dry BEC zone
D2-C7	1	94	Vegetated / Forested / Pure species stand / Deciduous-broadleaf or larch leading
D2-M2	1	94	Vegetated / Forested / Pure species stand / Deciduous-broadleaf or larch leading
D2-O1	1	1618	Vegetated / Forested / Recently burned (< 11 years) / Conifer forest / Crown closure <= 40% / Burned within 2-6 years
D2-S1	1	1621	Vegetated / Forested / Recently burned (< 11 years) / Mixedwood or deciduous / Burned within 2-10 years
M2-C7	1	1533	Vegetated / Forested / Mixed species stand / Not logged or logging > 6 years / 41-65% conifer / PY leading
M2-C7	1	122	Vegetated / Forested / Mixed species stand / Not logged or logging > 6 years / 41-65% conifer / FD leading / Not CWH, CDF or ICH BEC zone
M2-C7	1	166	Vegetated / Forested / Mixed species stand / Not logged or logging > 6 years / 65-80% conifer / FD leading / Not CWH, CDF or ICH (wet) BEC zone / Stand dense
O1-C7	1	96	Vegetated / Forested / Pure species stand / FD leading / Not logged or logging > 6 years / Species 1 HT >= 4m / Crown closure < 26% / Not CWH, CDF, MH or ICH (wet) BEC zone
O1-C7	1	1625	Vegetated / Non-forested / Recently burned (< 11 years) / Burned within 4-10 years
O1-M2	1	96	Vegetated / Forested / Pure species stand / FD leading / Not logged or logging > 6 years / Species 1 HT >= 4m / Crown closure < 26% / Not CWH, CDF, MH or ICH (wet) BEC zone
O1-M2	1	602	Vegetated / Non-forested / Not recently burned (> 10 years) / Logged (8-24 years) / Not CWH, MH or ICH (wet) BEC zone
S1-C3	1	310	Vegetated / Non-forested / Not recently burned (> 10 years) / Logged (<= 7 years) / Species 1 not P*, S*, B*, CW, YC, H*, FD
S1-C7	1	310	Vegetated / Non-forested / Not recently burned (> 10 years) / Logged (<= 7 years) / Species 1 not P*, S*, B*, CW, YC, H*, FD

See Perrakis et al. 2018 for decision tree and complete description of attributes, including species codes.
